# An Emerging Frontier in Intercellular Communication: Extracellular Vesicles in Regeneration

**DOI:** 10.3389/fcell.2022.849905

**Published:** 2022-05-11

**Authors:** Priscilla N. Avalos, David J. Forsthoefel

**Affiliations:** ^1^ Department of Cell Biology, College of Medicine, The University of Oklahoma Health Sciences Center, Oklahoma City, OK, United States; ^2^ Genes and Human Disease Research Program, Oklahoma Medical Research Foundation, Oklahoma City, OK, United States

**Keywords:** regeneration, tissue repair, extracellular vesicles (EVs), exosomes, intercellular communication, animal models

## Abstract

Regeneration requires cellular proliferation, differentiation, and other processes that are regulated by secreted cues originating from cells in the local environment. Recent studies suggest that signaling by extracellular vesicles (EVs), another mode of paracrine communication, may also play a significant role in coordinating cellular behaviors during regeneration. EVs are nanoparticles composed of a lipid bilayer enclosing proteins, nucleic acids, lipids, and other metabolites, and are secreted by most cell types. Upon EV uptake by target cells, EV cargo can influence diverse cellular behaviors during regeneration, including cell survival, immune responses, extracellular matrix remodeling, proliferation, migration, and differentiation. In this review, we briefly introduce the history of EV research and EV biogenesis. Then, we review current understanding of how EVs regulate cellular behaviors during regeneration derived from numerous studies of stem cell-derived EVs in mammalian injury models. Finally, we discuss the potential of other established and emerging research organisms to expand our mechanistic knowledge of basic EV biology, how injury modulates EV biogenesis, cellular sources of EVs *in vivo*, and the roles of EVs in organisms with greater regenerative capacity.

## 1 Introduction

Human tissues and organs are vulnerable to damage and degeneration caused by physical trauma, disease, and aging. Regenerative medicine seeks to develop therapeutic approaches to repair this damage, including through identification of ways to promote cellular behaviors required for successful regeneration (proliferation, differentiation, etc.), and to inhibit physiological responses to injury that hinder regeneration (excessive cell death, inflammation, fibrosis, etc.) (Iismaa et al., 2018). A growing body of research in many animal models has revealed that injury initiates a temporally and spatially coordinated series of events and cell behaviors, including wound closure, modulation of gene expression, immune responses, extracellular matrix (ECM) remodeling, re-establishment of polarity, proliferation, cell migration, and differentiation, that together lead to restoration of tissue form and function (Poss, 2010; Wells and Watt, 2018).

At each stage in the process of regeneration, molecules secreted by cells in the vicinity of the injury modulate these processes, controlling the molecular and physiological changes required for individual cells to collectively repair damaged tissue. Research in established models (e.g., fruit flies, frogs, zebrafish, and mice), as well as emerging models with greater regenerative capacity (e.g., hydra, planarians, salamanders, and African spiny mice), has demonstrated pro-regenerative roles for evolutionarily conserved growth factors, mitogens, cytokines, hormones, and morphogens [reviewed in Lucchetta and Ohlstein, 2012; Gemberling et al., 2013; McCusker et al., 2015; Reddien, 2018). Ongoing work has also identified novel secreted regulators of pro-regenerative proliferation, ECM modulation, and other processes (Kumar et al., 2007; Mokalled et al., 2016; Sugiura et al., 2016). Intercellular communication is thus likely to be a universal requirement for regeneration, suggesting that modulating cell:cell signaling could be a viable way to control human cells’ response to damage and improve regeneration.

Cells also communicate through the secretion of extracellular vesicles (EVs) that transport bioactive cargo between source and target cells, thereby modifying their behaviors (van Niel et al., 2018). The term “EV” broadly describes several classes of membranous nanoparticles secreted by cells in most (if not all) organisms including animals and plants, and even unicellular eukaryotes and prokaryotes (Edgar, 2016; Gurung et al., 2021). EVs possess a lipid bilayer that surrounds a lumen filled with cargo that can include proteins, RNA (mRNA, micro-RNA, long non-coding RNA, etc.), DNA, lipids, sugars, and metabolites (Kalluri and LeBleu, 2020). In animals, EVs are classified by several criteria. These include the cellular compartment from which they originate—exosomes are derived from the endosomal pathway, while microvesicles (MVs) or ectosomes are derived from the plasma membrane (PM)—as well as size, molecular composition and cargo, and method of purification (detailed further in Section 3) (van Niel et al., 2018). EVs isolated from biological fluids or produced by individual cell types are heterogeneous with respect to all of these criteria, thus, the development of methods to purify and define EV subclasses with specific activities is an ongoing priority (Bordanaba-Florit et al., 2021). Despite this complexity, dysregulation of EV biogenesis and function has been linked to numerous human pathologies, and efforts are underway to develop EVs as disease biomarkers and to engineer these vesicles for delivery of therapeutic cargo (Lener et al., 2015; Kalluri and LeBleu, 2020; Soekmadji et al., 2020).

EVs also promote tissue repair and regeneration. Stimulated by the initial discovery that EVs derived from mesenchymal stem cells (MSCs) could promote recovery from acute kidney injury (Bruno et al., 2009), hundreds of studies have now demonstrated EVs’ ability to protect against the deleterious effects of injury (e.g., ischemia) and to promote repair by modulating the hallmark cell behaviors required for regeneration (Jing et al., 2018; Tsiapalis and O’Driscoll, 2020). Below, we first briefly review the history of EV research and provide a broad overview of EV biogenesis. Then, we review selected studies of EVs in mammals, focusing on those that have demonstrated modulation of essential cellular behaviors and steps during regeneration, with an emphasis on studies that have identified specific cargo or signaling pathways likely to be responsible for EVs’ effects. Then, we highlight investigations of EV biology in other established models (zebrafish and fruit flies) and review evidence that EVs are produced by emerging research organisms with greater regenerative capacity (hydra, planaria, axolotls, and African spiny mice). Finally, we address how emerging models could help to address current knowledge gaps in EV biology and accelerate efforts to capitalize on the pro-regenerative potential of EVs.

## 2 Major Milestones in Extracellular Vesicle Research

**FIGURE 1:**
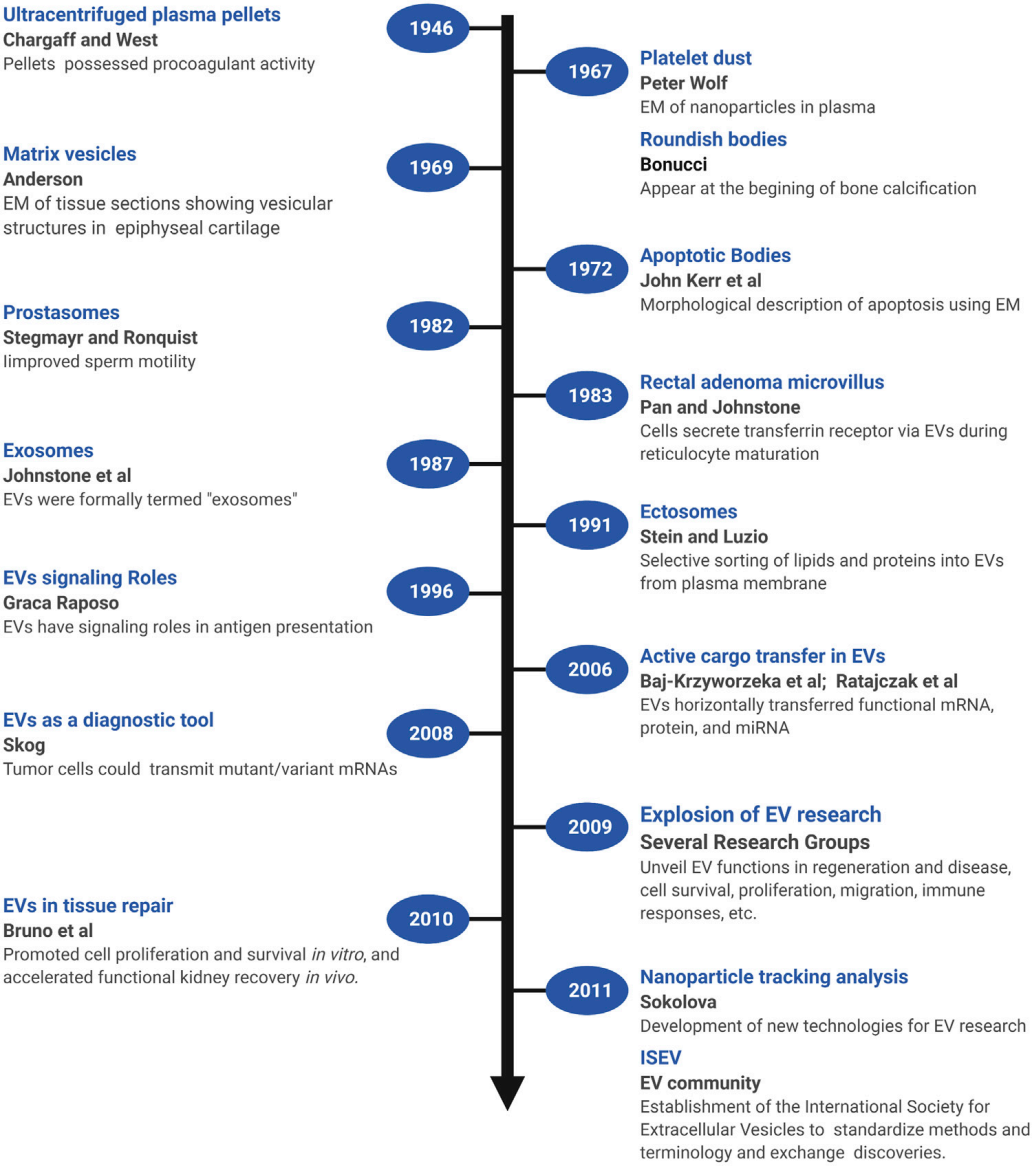
A brief timeline of EV research. Timeline of milestones in the investigation of EV biology and the roles of EVs in intercellular communication. Created with BioRender.com.

**FIGURE 2:**
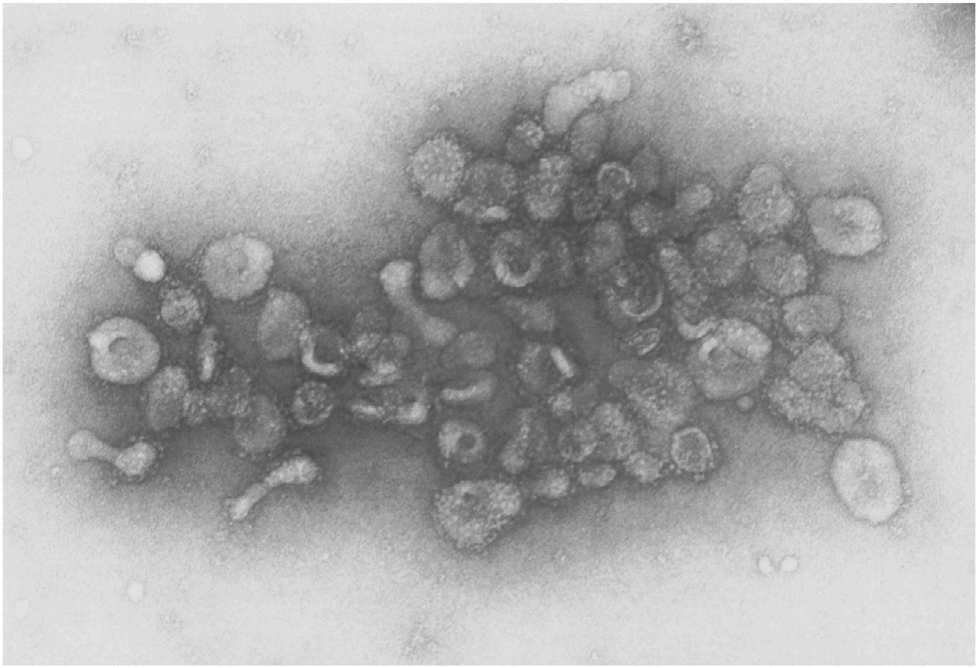
EVs from sheep reticulocytes. An early electron micrograph (123,000X) of EVs purified by Pan and Johnstone. Reprinted from Pan and Johnstone, “Fate of the Transferrin Receptor during Maturation of Sheep Reticulocytes *In Vitro*: Selective Externalization of the Receptor,” *Cell*, 33:967–977 (1983), with permission from Elsevier.

Several early observations suggested that cells might secrete membranous particles with biological activity (Figure 1). In 1946, Chargaff and West reported that pellets derived by ultracentrifugation from blood plasma possessed procoagulant activity (Chargaff and West, 1946). In 1967, Peter Wolf noted that coagulant activity of platelet-containing plasma and serum increases with storage over hours and was reduced by ultracentrifugation. Building on these observations, he isolated and directly observed particles that he called “platelet dust” in plasma using electron microscopy (EM) (Wolf, 1967). Bonucci and Anderson observed similar vesicular particles in the cartilage matrix during bone calcification (Bonucci, 1967; Anderson, 1969). Then, in the first detailed morphological description of apoptosis using EM, Kerr, Wyllie, and Currie described the production of apoptotic bodies (ABs, a type of EV derived from the plasma membrane of dying cells; see Section 3) during the process of “controlled cell deletion” (Kerr et al., 1972). This work was followed by other descriptions of similar vesicles from bat thyroid cells (Nunez et al., 1974), rectal adenoma microvillus cells (De Broe et al., 1975), and in other tissues and biological fluids (reviewed in Yáñez-Mó et al., 2015). Then, in the early 1980’s, several groups demonstrated that, during red blood cell maturation, the iron-trafficking protein transferrin and its receptor were transported to the “multivesicular endosome” (now called the multivesicular body or MVB), followed by subsequent secretion in EVs; these EVs were formally termed “exosomes” by Johnstone and colleagues in 1987 (Figure 2) (Pan and Johnstone, 1983; Harding et al., 1984; Johnstone et al., 1987). For decades, scientists had observed “membrane shedding” from the cell surface in response to various stimuli. In 1991, Stein and Luzio presented evidence for selective sorting of membrane lipids and proteins into plasma membrane-derived EVs secreted by complement-stimulated neutrophils (Stein and Luzio, 1991). They proposed the term “ectocytosis” for the release of “right-side out” vesicles where sorting of membrane components occurs to distinguish this mode of secretion from exocytosis.

Early on, EVs were proposed to play roles in disposing cellular waste or resisting complement attack during immune responses, but clues as to their functional roles in intercellular signaling were not recognized until later (Figure 1). In one of the first of these pioneering studies, Stegmayr and Ronquist reported that EVs secreted by prostate gland epithelium (which they termed “prostasomes”) improved sperm motility (Stegmayr and Ronquist, 1982). In 1996, Raposo and colleagues showed that EVs containing major histocompatibility complex II molecules secreted by B lymphocytes could stimulate IL-2 secretion by T cells, formally demonstrating that EVs could transfer biologically active molecules from one cell to another, and potentially play a long-range signaling role (Raposo et al., 1996). Beginning in 2006, multiple groups showed that EVs transferred functional mRNA, protein, and miRNA to recipient cells (Baj-Krzyworzeka et al., 2006; Ratajczak et al., 2006; Aliotta et al., 2007; Valadi et al., 2007). Tumor cells could also transmit mutant/variant mRNAs, suggesting the potential diagnostic utility of tumor-derived microvesicles (Skog et al., 2008). In 2009, building on observations that MSCs could promote tissue repair through secretion of soluble paracrine factors, Bruno and colleagues provided the first direct evidence that EVs could modulate tissue repair (Bruno et al., 2009). In a model of acute kidney injury, MSC-derived microvesicles promoted proliferation and survival of tubular epithelial cells *in vitro* and accelerated functional kidney recovery *in vivo* (Bruno et al., 2009). Subsequently, the field of EV research witnessed an explosion of effort to unveil the many functions of EVs in regeneration and disease, including hundreds of studies of the control of cell survival, immune responses, proliferation, migration, and other cellular processes (reviewed in Braicu et al., 2015; Pashoutan Sarvar et al., 2016; Jing et al., 2018; van Niel et al., 2018; Kalluri and LeBleu, 2020). Accompanying these advances, new technologies were also developed [e.g., dynamic light scattering (DLS), nanoparticle tracking analysis (NTA), and others] to quantify and characterize EVs from cultured cells and biological fluids (Sokolova et al., 2011; Shao et al., 2018). In addition, a broad coalition of investigators established the International Society for Extracellular Vesicles (ISEV, www.isev.org) in 2011 to help standardize methods and terminology (Witwer et al., 2013; Théry et al., 2018; Nieuwland et al., 2020), and facilitate the exchange of discoveries and approaches.

## 3 Extracellular Vesicle Biogenesis: Two Routes With Overlapping Mechanisms

**FIGURE 3:**
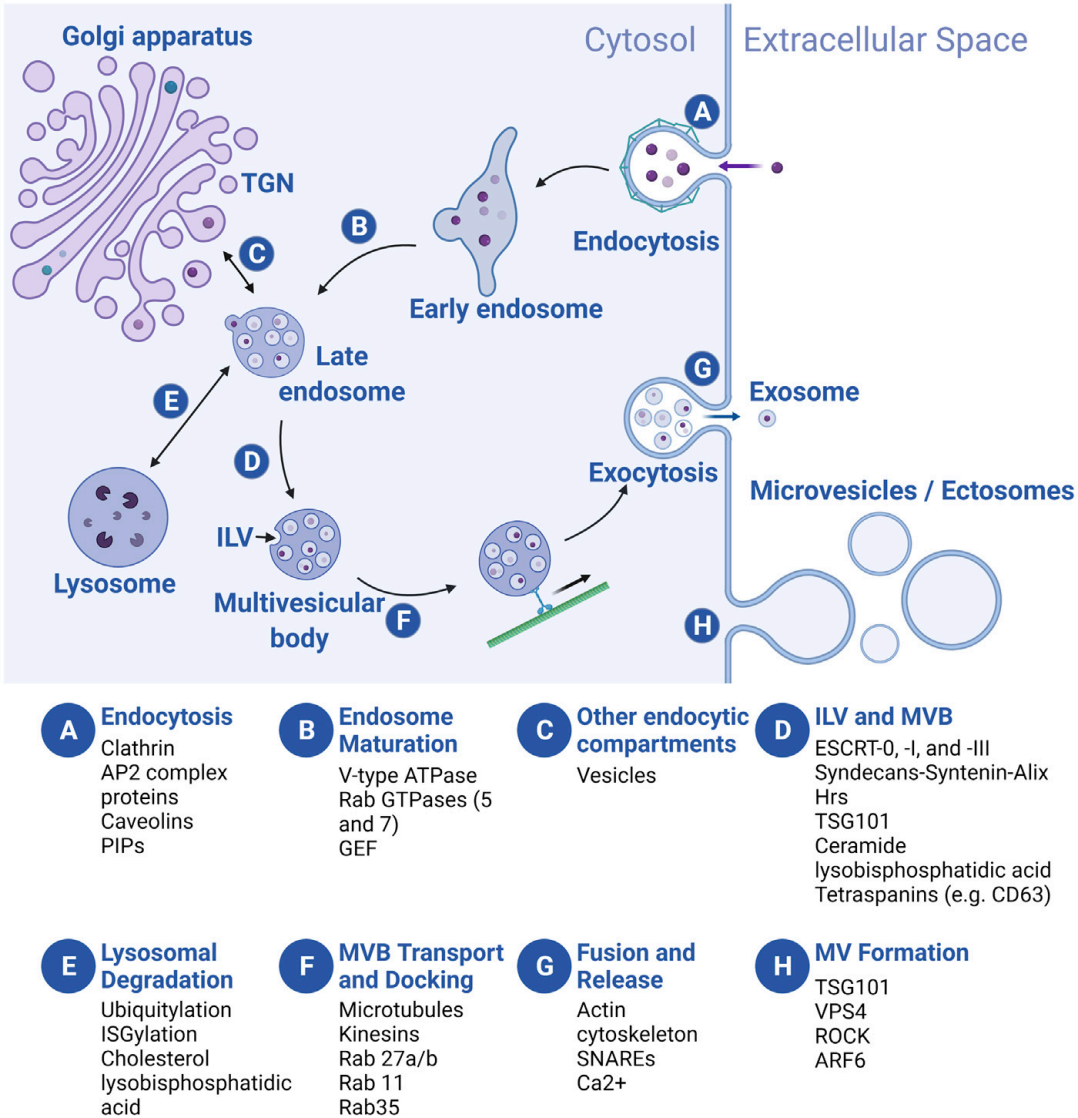
Overview of EV biogenesis. Letters in blue circles indicate steps in EV biogenesis. Regulators of each step are listed below. Exosome biogenesis begins with an endocytic event **(A)** that results in the formation of an early endosome (EE) which then matures into a late endosome (LE) **(B)**. During maturation, LEs receive cargo from several endocytic compartments such as the Golgi apparatus **(C)**, and cargo-filled vesicles bud internally (intraluminal vesicles, ILVs), creating the multivesicular body (MVB) **(D)**. MVBs are sorted to the lysosome for degradation **(E)**, or they traffic towards the plasma membrane (PM) **(F)** where they fuse and release the ILVs, now called exosomes **(G)**. Microvesicles (MVs) bud off directly from the PM **(H)**. For simplicity, we depict MVB formation following late endosome maturation but the MVB can de-attach from vesicular regions of both the early and late endosomes and ILVs can be added at multiple points along the pathway. TGN, trans-Golgi network. Created with BioRender.com.

EVs are derived from either the endosomal transport system or the plasma membrane (PM) (Figure 3) (Scott et al., 2014; van Niel et al., 2018; Kalluri and LeBleu, 2020). As discussed above, EVs from the endosomal pathway are exosomes or small EVs, while EVs generated from “right side out” budding of the PM are microvesicles (MVs) or ectosomes. MV subclasses include apoptotic bodies (ABs) that are produced by cells undergoing programmed cell death and large oncosomes secreted by cancer cells. Exosomes have a diameter between 30 and 150 nm, while microvesicles (50–1,000 nm), apoptotic bodies (500–2,000 nm), and oncosomes (up to 10 μm) are larger (van Niel et al., 2018). EVs are also commonly defined by characteristic cargo proteins, including Syntenin-1, ALG2-interacting protein X (ALIX), Tumor Suppressor Gene 101 (TSG101), Flotillin-1, and CD63, a member of the Tetraspanin family of transmembrane proteins, although identification of markers that distinguish exosome and microvesicle subclasses is an active area of investigation (Théry et al., 2018; Jeppesen et al., 2019; Kugeratski et al., 2021).

In the endosomal pathway, vesicles are initially derived from both clathrin-mediated and clathrin-independent endocytosis at the PM, often fusing with each other to form a tubular network of early endosomes (EEs) (Figure 3A). As vesicles mature into late endosomes (LEs), three processes occur: acidification of the vesicle lumen, recycling of some cargo back to the PM, and addition and loss of associated proteins involved in transport and other processes (e.g., Rab GTPases, see below) (Figure 3B). Membrane and cargo can also be delivered to EEs and LEs from the trans-Golgi network (TGN) (Figure 3C). During the maturation process, smaller vesicles invaginate into the EE and LE lumens, forming larger vesicles (multivesicular bodies or MVBs, also called multivesicular endosomes or MVEs) with smaller intraluminal vesicles (ILVs) inside (Figure 3D). At the LE, cargo molecules destined for degradation in the lysosome are segregated (based on ubiquitylation or ISGylation, see below) from those for secretion in exosomes. Subsequently, the LE/MVB can fuse with lysosomes for catabolism of its contents (Figure 3E), or be transported to the plasma membrane (Figure 3F). Upon fusion of the MVB with the PM, ILVs are released into the extracellular space as exosomes (Figure 3G). By contrast, MV formation is simpler: the PM buds outward, toward the extracellular space, followed by membrane scission to form MVs (Figure 3H). Regardless of cellular origin, the topology of both types of EVs is identical: extracellular domains of transmembrane proteins face the extracellular space, while vesicle lumens are equivalent to the cytoplasm and carry cytosolic cargo.

During exosome and MV production, conserved regulators of endocytosis, intracellular vesicle trafficking, and exocytosis play critical roles [reviewed in Scott et al., 2014; Elkin et al., 2016; Hessvik and Llorente 2018; Naslavsky and Caplan 2018; Palmulli and van Niel 2018; van Niel et al., 2018; Clancy et al., 2021; Gurung et al., 2021]. Here, we summarize widely studied regulators at each step of biogenesis. Exosome biogenesis begins at the PM, where clathrin and AP2 complex proteins coordinate vesicle endocytosis, in addition to clathrin-independent (e.g., caveolins and phosphatidyl inositols or PIPs) regulators (Figure 3A) (Scott et al., 2014). Following endocytosis, the Rab5 GTPase, an EE marker, promotes EE maturation to LEs by trafficking vacuolar (H+)-ATPases (V-ATPases) from the Golgi to endocytic vesicles and by recruiting the Rab7 GTPase, a LE marker that is required for trafficking to the lysosome (Figure 3B) (Naslavsky and Caplan, 2018). Next, ILV budding into the MVB is regulated by the ESCRT (endosomal sorting complexes required for transport) protein complexes (ESCRT-0, -I, and -III) and accessory proteins that recruit them (e.g., Syntenin and ALG-2-interacting protein X/ALIX) (Figure 3D) (Tamai et al., 2010; Baietti et al., 2012; Colombo et al., 2013). In addition, “ESCRT-independent” pathways for ILV formation likely exist, since ILVs still form in ESCRT-depleted cells (Stuffers et al., 2009). Transmembrane tetraspanin proteins (e.g., CD63), lysobisphosphatidic acid, and ceramide regulate cargo loading and membrane budding/scission in these pathways (Figure 3D) (Matsuo et al., 2004; Trajkovic et al., 2008; van Niel et al., 2011). Finally, MVBs are trafficked to the PM along microtubules by kinesins; MVB docking is coordinated by other Rab GTPases (e.g., Rab27a/b, Rab11, and Rab35) (Figure 3F), while MVB fusion with the PM is mainly regulated by vesicle-associated soluble *N*-ethylmaleimide-sensitive component attachment protein receptors (v-SNARES) and target membrane-associated t-SNARES (Figure 3G) (Saito et al., 1997; Savina et al., 2005; Fader et al., 2009; Hsu et al., 2010; Ostrowski et al., 2010; Ruiz-Martinez et al., 2016; Wei et al., 2017). At the PM, although many cells likely secrete exosomes constitutively, exosome secretion can be upregulated by stimuli such as cytoplasmic Ca^2+^ levels and extracellular cues (Savina et al., 2005; Savina et al., 2003; Fauré et al., 2006; Verweij et al., 2018). MV biogenesis occurs at the PM, rather than in the endosomal sorting pathway (Figure 3H) (Clancy et al., 2021). Nonetheless, some exosome biogenesis regulators (e.g., tumor susceptibility gene 101/TSG101, vacuolar protein sorting-associated protein 4/VPS4) also regulate biogenesis of MVs (Nabhan et al., 2012). Some authors suggest that unique regulators may also be involved, such as small GTPases thought to promote actomyosin contractility and membrane fission (e.g., RhoA/Rho-activated kinase/ROCK and ADP ribosylation factor 6/ARF6), and regulators of phospholipid and cholesterol distribution that may promote membrane curvature and cytoskeletal rearrangement (Del Conde et al., 2005; Lima et al., 2009; Muralidharan-Chari et al., 2009; Sedgwick et al., 2015), although debate exists (Ghossoub et al., 2014). Production of other PM-derived EVs such as large oncosomes and apoptotic bodies probably utilizes many of the same MV-related regulators of cytoskeleton and membrane lipid rearrangement, although similarities and differences in the biogenesis of different PM-derived EVs are not yet well understood (Atkin-Smith and Poon, 2017; Aoki et al., 2020; Clancy et al., 2021).

Two critical characteristics distinguish LEs destined to become exosomes: trafficking of the MVB to the PM rather than fusion with lysosomes, and loading of cargo into future exosomes as they bud into the MVB lumen as ILVs. How cells determine which MVBs to transport to the PM is not well understood, but recent studies have provided some clues. For example, cargo interactions with specific EV-associated molecules like ALIX and Tetraspanins (below) may steer MVBs towards secretion (Chairoungdua et al., 2010; Baietti et al., 2012; Hurwitz et al., 2016; Guix et al., 2017; Hurwitz et al., 2017). In addition, levels of specific lipids [e.g., high cholesterol (Möbius et al., 2002; Zimmerman et al., 2016) or low lysobisphosphatidic acid (White et al., 2006)] and reduced acidification of endosomes (van Weert et al., 1995; van Deurs et al., 1996; Liégeois et al., 2006) can reduce lysosomal targeting and shift MVB transport towards secretion, while post-transcriptional modification of MVB-localized proteins [e.g., ubiquitination (Buschow et al., 2005) and ISGylation (Villarroya-Beltri et al., 2016)] promote MVB degradation. For example, mutations in the E3 ubiquitin ligase Parkin or the ubiquitination site of the LE marker Rab7 (above) decreases MVB degradation and increases ILV formation, and exosome secretion (Song et al., 2016).

Cells also actively and selectively sort cargos (protein, mRNA, miRNA, ncRNA, lipids, etc.) into EVs, which are both enriched and depleted for specific molecules relative to their cells of origin (Valadi et al., 2007; Théry et al., 1999; Théry et al., 2001). Although regulation of selective cargo loading is not well understood, protein-protein, protein-lipid, RNA-protein, and even RNA-lipid interactions all play roles. Proteins can be targeted to EVs through interactions with tetraspanins (CD63, CD82, CD9, and CD81) (van Niel et al., 2011; Chairoungdua et al., 2010; Perez-Hernandez et al., 2013), chaperones (heat shock cognate 70 kDa protein/HSC70) (Géminard et al., 2004), ALIX (Baietti et al., 2012; Sun et al., 2019), and ADP ribosylation factor 6 (ARF6) (Muralidharan-Chari et al., 2009). Post-translational modifications like glycophosphatidylinositol (GPI) linkages (which promote membrane microdomain affinity) and farnesylation (which modulates protein-protein interactions) also promote EV targeting (Vidal et al., 1997; Rabesandratana et al., 1998; Luhtala et al., 2017). Numerous RNA binding proteins (RBPs) also regulate EV targeting of mRNA, miRNA, and other RNAs, including Argonaut 2 (AGO2), Y-Box 1 (YBX1), ALIX, and heterogenous nuclear nucleoproteins A2/B1 (HNRNPA2B1) (Villarroya-Beltri et al., 2013; Iavello et al., 2016; McKenzie et al., 2016; Shurtleff et al., 2016; Kossinova et al., 2017; Yanshina et al., 2018). In addition, specific RNA sequences (“EXOmotifs”) and secondary structures, post-translational RBP modification (e.g., sumoylation), and post-transcriptional RNA modification (e.g., 3′ uridylation) can mediate RNA-protein and possibly even RNA-lipid interactions to promote RNA targeting to EVs (Khvorova et al., 1999; Villarroya-Beltri et al., 2013; Koppers-Lalic et al., 2014; O’Brien et al., 2020). Cargo sorting primarily occurs during ILV formation for exosomes, and at the PM for MVs [reviewed in van Niel et al., 2018; Gurung et al., 2021]. However, regulation of vesicular trafficking elsewhere also influences cargo loading; for example, inhibition of transferrin receptor recycling back to the PM increases its abundance in exosomes (Vidal et al., 1997).

Once EVs are released from the cell surface, they are taken up by target cells, in which cargo must be trafficked properly to exert physiological effects. Binding to recipient cells is mediated by EV-bound integrins and other intercellular adhesion molecules (ICAMs) (Morelli et al., 2004), ECM components like fibronectin (Purushothaman et al., 2016), Tetraspanins (Rana et al., 2012), proteoglycans and glycoproteins (Bruno et al., 2009; Melo et al., 2015), and lipids (Toda et al., 2015; Matsumoto et al., 2017). Some EVs can influence target cells by directly binding PM receptors such as integrins or Toll-like receptors (Sobo-Vujanovic et al., 2014; Sung et al., 2015). However, for most cargos, cellular uptake is required and is mediated by most internalization mechanisms, including phagocytosis, macropinocytosis, direct fusion with the PM, and endocytosis mediated by clathrins, lipid rafts, and caveolins [reviewed in Gurung et al., 2021]. Once internalized, cargo can signal from the endosomal compartment (Shelke et al., 2019). More commonly, though, cargo enters the endocytic pathway, and then must escape degradation in lysosomes and enter the cytoplasm (for example, for miRNAs or mRNA to modulate gene expression). Although several mechanisms have been proposed (Gurung et al., 2021), the EV membrane may undergo a process of “back fusion” with the endosomal membrane, releasing contents into the cytosol (Joshi et al., 2020).

EV output, even from single cell types in culture, is highly heterogeneous. Although EV subtypes are likely to share common cargo, both exosomes and MVs can vary greatly in size, lipid composition, and levels and combinations of unique proteins, nucleic acids, and other metabolites (Colombo et al., 2013; Kowal et al., 2016; Willms et al., 2016; Kugeratski et al., 2021). EV heterogeneity arises, in part, from the multiple mechanisms that govern biogenesis and cargo loading, and because of the numerous intracellular locations at which these processes can be regulated. Additionally, most, if not all, known secretory pathway regulators have dual functions in EV biogenesis and intracellular trafficking, and few tools exist to target their functions or interactions at specific cellular locations or in subsets of intracellular vesicles. Cargo loading and MVB/ILV biogenesis can also be influenced by cellular state and environment (Segura et al., 2005; Carayon et al., 2011; Keller et al., 2020).

EV subtypes may have different functions, but the ability to purify, separate, and characterize them is still limited. For many years, ultracentrifugation (UC) has been a “gold standard” for total EV purification, but this method damages EVs (compromising their function), co-isolates contaminants (soluble proteins, lipoproteins, and endocytic vesicles), excludes smaller EVs, causes aggregation, and is time-consuming (Mol et al., 2017; Sidhom et al., 2020). Gentler polymer-based precipitation methods result in greater EV recovery, but can also co-purify contaminants (Zarovni et al., 2015; Rider et al., 2016; Weng et al., 2016; Brennan et al., 2020). Differential gradient centrifugation (DGC) can separate EVs from contaminants, but narrow density differences and the overlapping association of specific cargos with broad EV sizes limits DGC’s usefulness in characterizing heterogeneity (Kowal et al., 2016; Jeppesen et al., 2019). Combining methods like UC or precipitation with size exclusion chromatography also yields higher purity, despite sometimes lower yields of EVs within narrower size ranges (Sidhom et al., 2020). Immunoprecipitation-based approaches that target EV surface molecules like CD63 or phosphatidylserine are more selective (Nakai et al., 2016; Liangsupree et al., 2021), and transgenic affinity tagging enables purification of EV subtypes expressing specific proteins (Hung et al., 2018). Newer methods attempt to analyze EV preparations at the single-particle level, such as digital PCR, flow cytometry, and multiplexed immunolabeling, but their use remains limited due to expense and complexity (Hilton and White, 2021). Despite these advances, efforts to purify and define EV subclasses and the functional requirements for their biogenesis continue to be significant challenges, necessitating rigorous reporting to enable reproducibility and comparison (EV-TRACK Consortium et al., 2017; Veerman et al., 2021).

## 4 Extracellular Vesicles Promote Cellular Behaviors Required for Tissue Repair and Regeneration

In humans, many tissues undergo continuous cell replacement at high (e.g., blood, skin, intestine) or low (e.g., liver, lung, muscle) rates, in order to replace cells lost to normal physiological turnover or minor tissue damage (Iismaa et al., 2018). Organs and structures can be more severely damaged or lost after physical trauma, radiation, exposure to harmful chemicals or extreme temperatures, disease, and surgery. Unfortunately, though, humans possess limited ability to regenerate after these injuries, with only a few exceptions such as the liver and digit tips (Iismaa et al., 2018). This limited regenerative capacity is shared by widely studied human disease models, such as mouse, rat, and large mammals. By comparison, other animals (e.g., hydra, planaria, salamanders, zebrafish, and African spiny mouse) have greater regenerative capacity (Bely and Nyberg, 2010; Sánchez Alvarado, 2018). The regeneration observed throughout the animal kingdom suggests that it should be possible to modulate cellular and molecular mechanisms to improve tissue repair in mammalian models, and then to translate these approaches into regenerative therapies.

**FIGURE 4:**
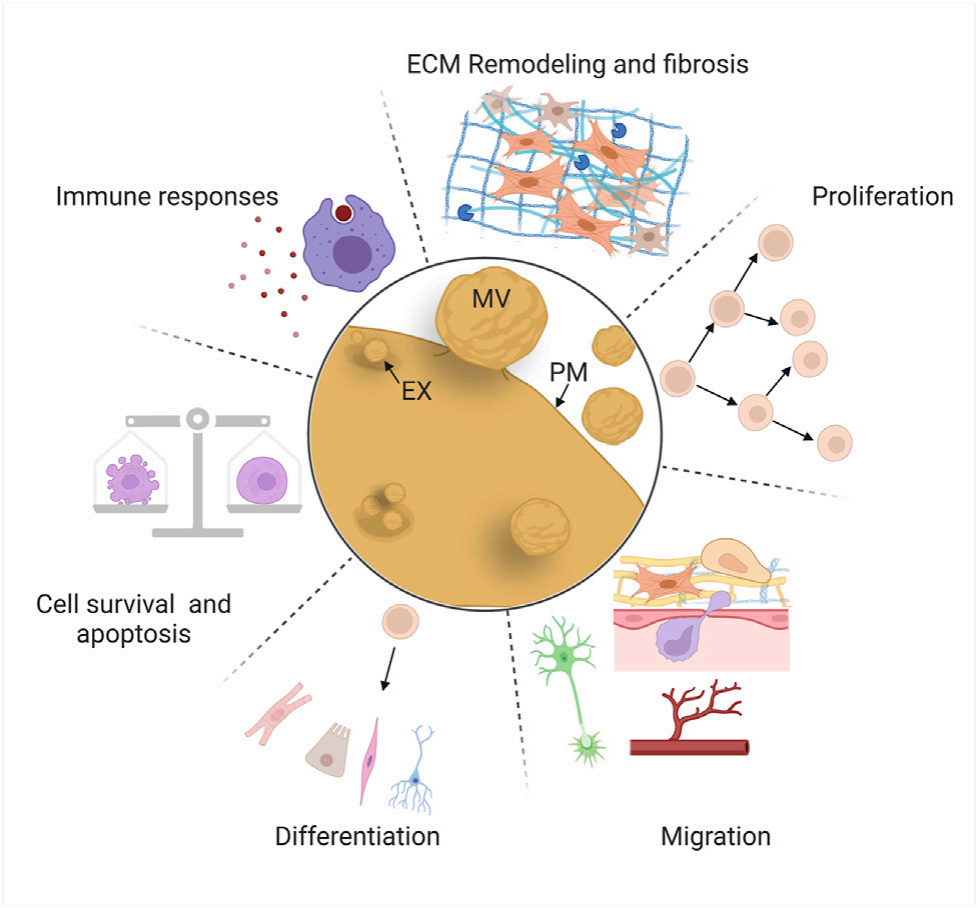
EV roles in mammalian repair and regeneration. EVs regulate “hallmark” cellular behaviors for successful regeneration: cell death and survival, immune responses, extracellular matrix remodeling, proliferation, migration, and differentiation. Schematics are stylized representations, and cells and structures are not drawn to relative scale. MV, microvesicle; EX, exosome; PM, plasma membrane. Created with BioRender.com.

Comparative studies have identified a set of “hallmark” cellular behaviors that must be coordinated to achieve successful regeneration, including cell death and survival, immune responses, extracellular matrix (ECM) remodeling, proliferation, migration, and differentiation (Figure 4) (e.g., 139, 140, 141). Most of these behaviors are initiated by wound signaling, when cells initiate signaling programs and transcriptional changes in response to local damage (Niethammer, 2016; Srivastava, 2021). These behaviors sometimes occur over broad sequential time windows, relative to injury, that tend to overlap with each other depending on the behavior, the cell type, and the context. One goal of regeneration research is to identify ways to modulate these cell behaviors to improve regenerative abilities by inducing reprogramming of cells to proliferative states or alternate fates (Srivastava and DeWitt, 2016), targeting inhibitory genes (Aguirre et al., 2014; Sekine et al., 2018), and introducing stem cells that produce new tissue and/or pro-regenerative cues (Kimbrel and Lanza, 2020).

Decades of basic research and translational efforts have focused on cell replacement therapy: the therapeutic introduction of MSCs (derived from a variety of tissues) or induced pluripotent stem cells (iPSCs, generated *ex vivo*) to counteract the effects of disease or tissue damage (Ullah et al., 2015; Kimbrel and Lanza, 2020). However, protective effects in multiple tissues (heart, blood vessels, and kidney) are often observed even in the absence of significant stem cell engraftment or survival, causing some investigators to explore whether paracrine factors could confer the bioactivity and benefits of the stem cells themselves (Lai et al., 2015; Gnecchi et al., 2016). These observations led to the investigation of EVs derived from a variety of stem cell types (MSCs, endothelial progenitor cells, cardiosphere-derived cells, lung spheroid cells, embryonic stem cells, and others) and the discovery that these vesicles protect against the consequences of injury (widespread cell death, fibrosis, etc.), or promote tissue repair (proliferation, migration, etc.) in *in vivo* and *ex vivo* models (Jing et al., 2018; Tsiapalis and O’Driscoll, 2020). In some cases, these studies have identified specific cellular behaviors affected by EVs and their cargos, and the cellular pathways that are modulated in recipient cells. Here, we review some of the most intriguing of these investigations, organized by the six major cellular behaviors we highlight above (Figure 4), focusing mainly on those in which likely mechanisms (e.g., specific cargos and molecular effects on recipient cells) have been identified.

### 4.1 Cell Death and Survival

One of the earliest consequences of acute tissue damage is increased cell death through apoptosis, necrosis, and other mechanisms (Pellettieri et al., 2010; Guerin et al., 2021). For example, mechanical damage to the spinal cord induces neuronal death, while after stroke or heart attack, cells die due to reduced blood supply and hypoxia (ischemia) (Konstantinidis et al., 2012; Şekerdağ et al., 2018; Shi et al., 2021). Although restoration of blood flow (reperfusion) is required to prevent further cell death and support regeneration, it initially exacerbates damage by causing elevated production of reactive oxygen species, oxidative stress, reduced nitric oxide levels, and inflammation (“ischemia-reperfusion injury”) (Wu et al., 2018). Elevated cell death also occurs in chronic organ disease, and can be catastrophic in acute organ failure due to the loss of functional tissue (Linkermann et al., 2014; Luedde et al., 2014; Sauler et al., 2019). Dying apoptotic cells can induce further cell death in nearby cells non-autonomously, extending tissue damage (Pérez-Garijo et al., 2013). Promoting survival of endogenous cells after acute injuries, or of therapeutically grafted stem cells, can improve tissue repair and is, therefore, one key goal of regenerative medicine (Abdelwahid et al., 2016; Hilton et al., 2017).

Bruno and colleagues reported one of the first examples of a pro-survival role for EVs after tissue damage in a model of acute kidney injury (AKI), after which MSC-derived EVs promoted survival of tubular epithelial cells *in vitro*, and accelerated functional kidney recovery *in vivo* (Bruno et al., 2009). Subsequently, several groups identified EV-transported miRNAs with anti-apoptotic activity in AKI. miR-486-5p (which targets the phosphatase and tensin homolog, PTEN) from endothelial colony-forming cell (ECFC) EVs reduces apoptosis after ischemia/reperfusion injury, and miR-21 (which targets numerous tumor suppressors including PTEN), possibly derived from skeletal muscle EVs, promotes renal tubular epithelial cell survival after sepsis-induced AKI (Viñas et al., 2016; Pan et al., 2019; Viñas et al., 2021). In another example, cardiosphere-derived EVs promote functional recovery in a mouse model of myocardial infarction (MI), and neonatal rat cardiomyocyte (CM) survival *in vitro* (Ibrahim et al., 2014). These effects are mediated by miR-146a, which downregulates interleukin-1 receptor-associated kinase (Irak1) and tumor necrosis factor receptor-associated factor 6 (Traf6), effectors of Toll-like receptor signaling (Ibrahim et al., 2014). In the CNS, systemic administration of MSC-derived EVs improves functional recovery and reduced apoptosis in a rat model of spinal cord injury, in part by elevating expression of the anti-apoptotic protein B-cell lymphoma 2 (Bcl-2) and decreasing expression of the pro-apoptotic protein Bcl-2-associated X protein (Bax); future work will be required to identify the EV cargo responsible for this effect (Huang et al., 2017).

Additional reports of EVs that promote cell survival in both *in vivo* and *in vitro* injury models exist, although the precise mechanisms by which these EVs act are not as well understood (Wu et al., 2021). EVs also promote apoptosis, for example by transporting Caspase-1 and Gasdermin D from monocytes to pulmonary vascular endothelial cells in an *in vitro* model of acute lung injury (Mitra et al., 2018). By contrast, dying cells can also promote damage-induced proliferation, and apoptotic cells release EVs that probably play additional signaling roles (Chera et al., 2009; Brock et al., 2019; Kakarla et al., 2020). These complexities suggest that efforts to promote cell survival by controlling EV activity will need to be informed by a detailed understanding of their context-specific roles.

### 4.2 Immune Responses and Inflammation

Tissue damage stimulates the recruitment and activation of innate and adaptive immune cells with functions in host defense, debris clearance, and coordination of other cells’ roles in regeneration (Godwin et al., 2017a; Julier et al., 2017; Abnave and Ghigo, 2019). Neutrophils and macrophages are innate immune cells with prominent early roles during repair and regeneration (Wynn and Vannella, 2016; Wang, 2018). Immediately after injury, pathogen-associated molecular patterns (PAMPs) and damage-associated molecular patterns (DAMPs, for example, heat shock proteins and histones released by damaged tissue) are sensed by tissue-resident macrophages, which then secrete chemoattractants and pro-inflammatory cytokines to recruit circulating neutrophils and monocytes (Julier et al., 2017). Next, neutrophils that infiltrate the tissue secrete cytokines to amplify the inflammatory response by recruiting and activating other immune cell types, as well as antimicrobial compounds, proteases, and reactive oxygen species (ROS) to kill invading pathogens (Wang, 2018). Then, macrophages fulfill early pro-inflammatory roles by clearing bacteria, necrotic cells, apoptotic neutrophils, and debris; later, macrophages adopt pro-regenerative roles by terminating inflammation, promoting proliferation and differentiation of MSCs, and stimulating ECM remodeling by fibroblasts and myofibroblasts (Abnave and Ghigo, 2019; Wynn and Vannella, 2016). Recent studies also implicate adaptive immune cells in regeneration. For example, regulatory T cells (Tregs) promote macrophage polarization towards pro-reparative states (the “M1-to-M2” transition), and specialized tissue-resident γδ T cells that reside in surface epithelia secrete pro-inflammatory chemokines and pro-repair growth factors (Julier et al., 2017; Abnave and Ghigo, 2019; Ramirez et al., 2015). Immunity-related phenotypes in mouse and human illustrate the importance of immune modulation during tissue repair. For example, protozoan infection inhibits muscle regeneration by decreasing Tregs and increasing pro-inflammatory macrophages (Jin et al., 2017), while in the liver, repeated acute injury and autoimmune diseases can cause persistent activation of macrophages, hepatic myofibroblasts and stellate cells, inhibiting repair and functional recovery (Pellicoro et al., 2014). Also, chronic injury and inflammation cause fibrosis and scarring in multiple organs (discussed further in Section 4.3) (Mack, 2018).

Immune cells produce EVs with both pro- and anti-regenerative activity (Wang et al., 2020). For example, Hervera and colleagues found that macrophage-derived EVs deliver NADPH oxidase 2 (NOX2) to damaged dorsal root ganglion neurons, promoting PTEN oxidation, activation of Akt signaling, neurite outgrowth, and recovery after sciatic nerve crush injury (Hervera et al., 2018). In the mouse intestine, macrophages secrete Wnt packaged in EVs to promote intestinal stem cell survival and recovery from radiation-induced injury (Saha et al., 2016). EVs from immune cells can also negatively impact regeneration. For example, Slater et al. (2017) found that neutrophil-derived EVs transport myeloperoxidase, a potent antimicrobial enzyme that also induces oxidative tissue damage; these EVs inhibit healing of the wounded colonic mucosa in mice by preventing intestinal epithelial cell spreading and proliferation. Recent *in vitro* studies suggest macrophage-derived EVs promote osteogenesis (Liu et al., 2020; Li et al., 2021a). However, in an interesting example of how disease can dysregulate EV activity, Zhang et al. (2021a) found that EVs secreted by bone marrow-derived rat macrophages from diabetic mice impair osteogenic differentiation of bone marrow stem cells and compromise femoral fracture healing, as compared to EVs from healthy animals. EVs from diabetic rats possess high levels of the Smad1-targeting miR-144-5p, negatively inhibiting pro-osteogenic bone morphogenetic protein (BMP) signaling (Zhang et al., 2021a).

EVs from stem cells possess immunoregulatory potential, and influence the functions of most immune cell types, demonstrating that immune cells are also EV recipients during regeneration (Xie et al., 2020). For example, Li et al. (2016) found that EVs derived from human umbilical cord MSCs (HU-MSCs) suppress inflammation in a rat burn injury model by lowering inflammatory cytokine levels (TNF-α and IL-1β), reducing the number of neutrophils and macrophages, and increasing the levels of anti-inflammatory IL-10. The authors attributed the mechanism to EV-derived miR-181c, which downregulates pro-inflammatory TLR4 signaling through the NF-κB/P65 pathway. In another study, HUC-MSC-derived EVs inhibit the injury-induced accumulation of natural killer (NK) cells, thereby protecting against renal injury in a rat model of ischemia-reperfusion injury (Zou et al., 2016).

These studies demonstrate that EV signaling is likely to occur bidirectionally between immune cells and other cell types in injured tissue and that signaling can either assist or impair regeneration. The timing and intensity of immune cell responses, and the diversity of cell states adopted by macrophages and other immune cell types, vary across tissues and in response to different types of injury (Wynn and Vannella, 2016; Godwin et al., 2017a; Julier et al., 2017). Thus, two important challenges are to refine our understanding of context-dependent mechanisms that may control EV biogenesis during immune responses and to continue identifying cargo with spatial, temporal, and cell-type specific roles in immunoregulation.

### 4.3 Extracellular Matrix Remodeling and Fibrosis

The extracellular matrix (ECM) is composed of collagen, fibronectin, elastin, proteoglycans, and other molecules that play structural roles in the organization of tissue architecture. ECM also serves as a substrate for cellular migration, and as a reservoir of signaling molecules that regulate activities of numerous cell types (Rozario and DeSimone, 2010; Godwin et al., 2014). During regeneration, after initial formation of a temporary fibrin-based clot, immune cells, fibroblasts, myofibroblasts, and other cell types degrade some ECM molecules (by secreting matrix metalloproteinases and other enzymes) and deposit new ECM, gradually remodeling the matrix in sequential steps as repair proceeds. Although specific combinations of cell types and matrix molecules during ECM remodeling vary by tissue [reviewed in Godwin et al., 2014; Xue and Jackson, 2015], ECM remodeling generally facilitates clearance of damaged tissue, proliferation and differentiation of progenitor cells, and migration and assembly of cells into new tissue. During regeneration of some mouse and human tissues (e.g., liver), ECM remodeling is often coordinated effectively (Cordero-Espinoza and Huch, 2018). More commonly, fibrosis and scarring (deposition of a fibrotic ECM matrix) are the default outcome after cutaneous wounds, spinal cord injury, ischemic heart and kidney damage, etc., especially with chronic inflammation (Leoni et al., 2015; Mack, 2018; Willis et al., 2018). Fibrosis occurs primarily due to the differentiation and persistence of myofibroblasts in granulation tissue (formed after initial clotting), which occurs in response to growth factors secreted by monocytes and other cells (Darby and Hewitson, 2007; Godwin et al., 2014; Julier et al., 2017). Myofibroblasts secrete a dense matrix of collagen (the fibrotic scar) that is not resolved, inhibiting regeneration and compromising normal organ function (Darby et al., 2016; Willis et al., 2018). Other ECM-secreting cells also inhibit regeneration; for example, nervous system glia (microglia and astrocytes) deposit excessive chondroitin sulfate proteoglycans, forming a glial scar that prevents spinal cord regeneration (Yang et al., 2020). By contrast, animals with greater regenerative capacity achieve scar-free healing and regeneration by restricting accumulation of pro-inflammatory immune cells and pro-fibrotic myofibroblasts, by promoting recruitment of pro-repair/anti-fibrotic macrophages, or by resolving fibrotic matrix over time (Lévesque et al., 2010; González-Rosa et al., 2011; Seifert et al., 2012; Godwin et al., 2013; Richardson et al., 2013; Godwin et al., 2017b; Simkin et al., 2017). In addition, the developing human fetus also heals wounds without scarring, possibly due to differences between adult and fetal fibroblast ECM deposition (Lorenz et al., 2003). Together, these observations suggest that therapeutic control of ECM remodeling might be possible to achieve scar-free adult tissue regeneration.

EVs attenuate differentiation and activity of ECM-producing cells, reducing fibrosis in injury models. For example, EVs derived from human adipocyte stem cells (hASC-EVs) promote ECM remodeling and scarless healing of dorsal skin incisions in mice by inhibiting myofibroblast differentiation and increasing the ratios of collagen III to collagen I and TGFβ-3 to TGFβ-1, similar to levels in fetal scarless wound healing (Wang et al., 2017). hASC-EVs also reduced hypertrophic scarring during wound healing in rabbit ears, by suppressing myofibroblast differentiation and collagen deposition (Zhu et al., 2020). In an example of EVs’ therapeutic potential, Dinh and colleagues showed that inhalation of lung spheroid cell-derived EVs inhibits collagen deposition and improves alveolar repair in mouse and rat models of pulmonary fibrosis, possibly by transporting miR-30a, an anti-fibrotic miRNA, to matrix-secreting cells (Berschneider et al., 2014; Dinh et al., 2020). In pig models of myocardial infarction (MI), delivery of EVs from cardiosphere-derived cells reduces collagen deposition, cardiac hypertrophy, and scarring, although the precise mechanism remains to be uncovered (Gallet et al., 2017). In the nervous system, EVs from anti-inflammatory M2 microglia inhibit astrocyte proliferation and glial scarring in a mouse stroke model, by transporting miR-124 to downregulate signal inducer and activator of transcription 3 (STAT3), a known promoter of astrogliosis and scarring (Herrmann et al., 2008; Li et al., 2021b).

EVs secreted by cells in injured organs are also likely to exacerbate fibrosis (Brigstock, 2021). For example, ischemia-reperfusion injury increases EV secretion by mouse kidney tubular epithelial cells; inhibiting EV biogenesis by knocking out Rab27a reduces EV secretion, fibronectin levels, and renal fibrosis *in vivo* (Zhou et al., 2021). Inhibition of miR-150-5p, which is enriched in EVs from cultured hypoxic tubular cells and targets suppressor of cytokine signaling 1 (SOCS1), reduces fibroblast activation, fibronectin expression, and fibrosis *in vivo,* demonstrating that hypoxic tubular cells secrete EVs that aggravate renal fibrosis (Zhou et al., 2021). In addition, EVs secreted by fibrotic kidneys or from hypoxic cultured tubular epithelial cells are enriched for TGF-β1 mRNA, which induces fibrosis in murine kidneys, and promotes fibroblast activation and collagen secretion (Borges et al., 2013). In a second example, hypoxic and angiotensin II-treated cardiomyocytes (CMs) secrete EVs enriched for miR-208a, which promote proliferation and differentiation of cultured fibroblasts into collagen-secreting myofibroblasts (Yang et al., 2018). Inhibition of miR-208a reduces MI-induced fibrosis, while injection of miR-208a-containing EVs into post-MI rat hearts increases fibrosis, most likely by targeting mRNA encoding Dual specificity tyrosine-phosphorylation-regulated kinase 2 (*Dyrk2*), an inhibitor of nuclear factor of activated T-cells (NFAT)-mediated myofibroblast differentiation (Yang et al., 2018). These data demonstrate that MI also induces production of pro-fibrotic EVs.

Recently, EVs called matrix-bound nanovesicles (MBVs) were identified in ECM bioscaffolds from decellularized tissue used as biomaterials to promote tissue repair after surgery (Huleihel et al., 2016). Subsequent work suggests that MBVs confer at least some of the pro-regenerative activities of bioscaffolds (inflammation modulation, cell survival, neurite extension, etc.) (Huleihel et al., 2016; Huleihel et al., 2017; van der Merwe et al., 2019) and that the lipid and nucleic acid profile of MBVs is unique compared to liquid-phase EVs (Hussey et al., 2020). Whether these MBVs serve as spatially restricted signals or as a “reservoir” of cues that can be released upon ECM remodeling, or whether they have other roles, are open questions (Lewin et al., 2020). To summarize, EVs appear to regulate ECM remodeling in both beneficial and detrimental ways during regeneration, and ECM also may reciprocally regulate the activity or localization of some EVs/MBVs. Delivery or inhibition of anti- or pro-fibrotic EVs, respectively, as well as modulation of interactions between ECM and EVs are all potentially viable ways to fine-tune ECM remodeling, minimize scarring, and improve regeneration.

### 4.4 Cellular Proliferation, De-differentiation, and Pluripotency

Cellular proliferation, the process by which a cell divides and produces two daughter cells, is essential for the regeneration of new tissue (Tanaka and Reddien, 2011). While the source and differentiation potential of cycling cells varies widely across organs, tissues, and animals, injury almost universally stimulates proliferation (Ricci and Srivastava, 2018). Proliferation of many types of stem and progenitor cells is required to produce progeny that rebuilds lost and damaged tissue. For example, fibroblast proliferation is required for ECM remodeling (Plikus et al., 2021); endothelial cell proliferation is required to revascularize regenerating tissue (Pecoraro et al., 2021); hepatocytes proliferate to rebuild liver mass (Chen et al., 2020); and multiple cell types proliferate after acute and chronic lung injury (Kotton and Morrisey, 2014). In mammals, injury increases proliferation through a variety of mechanisms, including by stimulating division of tissue-resident stem cell populations (Hsu and Fuchs, 2021); promoting cell cycle re-entry of quiescent stem cells (Fu et al., 2015); activating facultative stem cells that normally exist in a fully differentiated state (Leach and Morrisey, 2018); and expanding rare injury-responsive subpopulations (Wilson et al., 2008; Ayyaz et al., 2019). Because depletion of stem and progenitor cells would compromise regeneration, proliferation must also balance renewal of the pool of cycling cells and maintenance of their pluripotency with production of post-mitotic progeny (discussed in Section 4.6) (Feige et al., 2018; Gehart and Clevers, 2019). Identifying ways to induce or elevate proliferation in response to damage could help to promote repair in less injury-responsive tissues, and to control proliferation more precisely in specific injury contexts.

The first evidence that EVs promote cell proliferation came from *in vitro* immunology studies. Raposo and colleagues observed that T cells incubated with B-cell-derived EVs proliferated as a response to antigen presentation (Raposo et al., 1996). More recently, EVs have been shown to regulate proliferation of many cell types in various tissue damage models (Jing et al., 2018; Roefs et al., 2020; Tsiapalis and O’Driscoll, 2020). For example, Nojima et al. (2016) found that hepatocyte-derived EVs promote both hepatocyte proliferation and mouse liver regeneration *in vivo* after injury caused by both ischemia-reperfusion and partial hepatectomy (Nojima et al., 2016). This effect is mediated by the transfer of ceramide, neutral ceramidase, and sphingosine kinase 2, enabling hepatocytes to produce intracellular sphingosine-1-phosphate to stimulate proliferation (Nojima et al., 2016). In another example, amniotic fluid stem cell derived-EVs attenuate intestinal injury in a mouse model of necrotic enterocolitis by activating the Wnt signaling pathway, which increases proliferation *in vivo* leading to regeneration of intestinal epithelium (Li et al., 2020).

EVs also stimulate proliferation of cell types that normally do not respond to injury. For example, EVs derived from cardiac explant-derived progenitor cells carrying Periostin induce cell-cycle re-entry and proliferation by neonatal rat CMs both *in vitro* and *in vivo*, and by adult rat CMs after MI, through a focal adhesion kinase (FAK) and Yes-associated protein (YAP) signaling pathway (Balbi et al., 2021). EVs also influence the pluripotency and plasticity of proliferative cells. For instance, fibronectin associated with embryonic stem cell (ESC)-derived EVs engaged integrins and stimulated FAK activation in ESCs cultured in differentiation-promoting media; this maintains pluripotency *in vitro* and preserves the ability of EV-treated cells to generate chimeric mice (Hur et al., 2021). In another example, EVs from gingiva-derived MSCs promote recovery from peripheral nerve crush in mice by increasing Schwann cell dedifferentiation/activation, proliferation, and migration through c-JUN N-terminal kinase (JNK) signaling (Mao et al., 2019).

Several groups have extended studies of EVs’ roles in proliferation even further by engineering custom EVs with mitogenic activity. For example, Staufer and colleagues engineered fully synthetic EVs, identifying minimal protein and miRNA cargo required to promote proliferation of keratinocytes (Staufer et al., 2021). Wang and colleagues engineered MSCs to produce EVs tagged with a short peptide enabling their targeting to extracellular cardiac troponin I, which is released by necrotic and apoptotic cells during MI (Wang et al., 2018). When these EVs were loaded with the pro-proliferative *H. sapiens* hsa-miR-590-3p and introduced into a rat MI model, they promoted CM proliferation and improved heart function (Wang et al., 2018). Altogether, these studies demonstrate that EVs can promote proliferation, de-differentiation, and stemness during mammalian regeneration, and provide evidence that EVs could be engineered to perform similar therapeutic roles in human patients.

### 4.5 Cell Migration, Angiogenesis, and Neurite Growth

Individual cells migrate to facilitate multiple steps of regeneration. For example, fibroblasts migrate to remodel the ECM (Plikus et al., 2021), immune cells extravasate from the blood supply to promote inflammation and clear microbes (Julier et al., 2017), muscle satellite cells migrate to repair damaged muscle (Choi et al., 2020), and MSCs migrate to generate new cartilage, bone, fat, and other tissues (de Lucas et al., 2018). Cells also migrate collectively (Friedl and Gilmour, 2009): epithelial cells migrate in sheets underneath fibrin clots to re-epithelialize cutaneous injuries (Shaw and Martin, 2009), and endothelial cells migrate collectively during angiogenic sprouting and revascularization of new tissue (Pecoraro et al., 2021). Migration is stimulated by environmental cues (e.g., chemokines) as well as mechanical forces, and requires cytoskeletal rearrangements and modulation of cell:cell and cell:matrix interactions (Trepat et al., 2012; Shellard and Mayor, 2020). Often, migration occurs at multiple time points during regeneration and is required for subsequent cell behaviors and steps. For example, hypoxia in injured tissues stimulates angiogenesis; this provides nutrients and oxygen, and also enables migration of immune cells that regulate inflammation and stem cells that proliferate and differentiate into new tissue (Pugh and Ratcliffe, 2003; Julier et al., 2017; de Lucas et al., 2018). After peripheral nerve transection, hypoxia stimulates macrophages to promote the growth of new blood vessels; these serve as substrates for migrating Schwann cells that subsequently guide axons’ regrowth across the cut site back to their targets (Cattin et al., 2015). Because cell migration is vital for regeneration, researchers have sought ways to control and engineer cell movement to improve tissue repair (Shin et al., 2020; Shim et al., 2021).

EVs promote migratory cell behaviors during regeneration. Cooper et al. (2018) found that EVs from human adipose-derived stem cells transport the lncRNA *metastasis-associated lung adenocarcinoma transcript 1* (*MALAT1*, a miRNA sponge) to promote migration of human dermal fibroblasts *in vitro*, and ischemic skin wound healing in a rat model. In mice, acute lung injury upregulates biogenesis of EVs carrying miRNA-17 and miRNA-221, which increases macrophage migration and lung infiltration by promoting Integrin β1 recycling to the plasma membrane (Lee et al., 2017). Platelet-derived microparticles transfer the chemokine receptor CXCR4 to angiogenic early outgrowth cells (EOCs), promoting their cytoskeletal rearrangement and migration *in vitro*, and improving transplanted EOC adhesion and re-endothelialization in a mouse model of carotid artery injury (Mause et al., 2010). EVs from mechanically stimulated Schwann cells transfer miR-23b-3p to dorsal root ganglion neurons, targeting the repulsive axon guidance protein Neuropilin 1 to enhance neurite outgrowth *in vitro* and rat sciatic nerve regeneration *in vivo* (Xia et al., 2020). EVs can also inhibit cell migration. For example, EVs from bone MSCs inhibit migration of vascular pericytes *in vitro via* NF-κB p65 signaling, and reduce vascular permeability after spinal cord injury in rats, improving integrity of the brain-spinal cord barrier (Lu et al., 2019).

In addition to being an EV target, migrating cells also secrete EVs with adhesive, chemotactic, and other characteristics. For example, autocrine EV secretion by cancer cells promotes motility, adhesion, and directional migration (Sung et al., 2015), and fibrosarcoma cells deposit an “exosome trail” that functions in a paracrine manner as a migration “track” for follower cells (Sung et al., 2020). In addition, Ma and colleagues discovered a large EV called the “migrasome” that is released from retraction fibers at the rear of migrating fibroblasts, keratinocytes, and cancer cells (Ma et al., 2015a). Migrasomes guided cell migration *in vivo* during zebrafish organogenesis*,* and transfer mRNA and protein, although only a few active cargo molecules have been identified so far (Jiang et al., 2019; Zhu et al., 2021). Whether exosome trails and/or migrasomes might function in paracrine regulation of cell migration during tissue repair remains to be investigated.

### 4.6 Differentiation

Differentiation is essential for regeneration: as progeny of stem and progenitor cells assemble into tissues and organs, they also must specialize for individual physiological roles. For example, satellite cells differentiate into muscle fibers after damage (Collins et al., 2005); hematopoietic stem cells differentiate into mature blood cells after hemorrhage and sepsis (Kelly et al., 2021); and stem cell-derived transit-amplifying cells in the epidermis and intestinal crypt differentiate to replace damaged epithelia (Blanpain and Fuchs, 2014). Other cell types differentiate to fulfill more transient but required roles: fibroblasts differentiate into myofibroblasts in response to injury to close cutaneous wounds and remodel the ECM (Plikus et al., 2021), and monocytes differentiate into macrophages at the injury site to phagocytose pathogens and secrete chemokines (Wynn and Vannella, 2016). The lineage potential of proliferating cells varies by tissue; for example, muscle satellite cells will give rise only to muscle, while intestinal stem cells give rise to absorptive, secretory, endocrine, and immune cells. Even so, the stages of differentiation and molecular mechanisms guiding these steps are broadly similar. A cell’s initial decision to terminally differentiate (“fate specification”) is often linked to withdrawal from the cell cycle (Dalton, 2015; Soufi and Dalton, 2016; Zhao et al., 2020). Subsequently, chromatin modifications and changes in gene expression drive commitment and morphogenesis (Myster and Duronio, 2000; Ma et al., 2015b; Soufi and Dalton, 2016). Controlling differentiation could improve regeneration by increasing the production of missing tissue, or by reducing the presence of cells with inhibitory activities.

EVs influence differentiation in tissue repair models (Tsiapalis and O’Driscoll, 2020; Roefs et al., 2020). For example, osteoclast-derived EVs carrying miR-324 promote MSC differentiation into osteoclasts and mineralization by inhibiting ARHGAP1, a negative regulator of osteogenesis (Liang et al., 2021). When seeded into a decalcified bone matrix and grafted into a mouse calvarial defect model, miR-324 carried by EVs promotes bone regeneration (Liang et al., 2021). Articular chondrocyte EVs promote differentiation of HUC-MSCs into chondrogenic cells (possibly *via* activation of autophagy) and accelerate cartilage regeneration in rabbits with a knee joint cartilage defect (Ma et al., 2020). Dental pulp cell-derived EVs induce differentiation of human dental pulp stem cells (DPSCs) into odontoblasts *in vitro* and *in vivo* by activating p38 MAPK signaling and promote dental pulp-like regeneration in a mouse *in vivo* tooth root slice model (Huang et al., 2016). Similarly, EVs from Hertwig’s epithelial root sheath cells induce odontogenic differentiation of dental papilla cells (DPC) and promote formation of dental pulp-like tissue that is both vascularized and innervated, possibly by activating Wnt/β-catenin signaling (Zhang et al., 2020). EVs from adipose tissue promote adipose differentiation from human adipose-derived stem cells (HASCs), suggesting the potential to supply soft tissue replacements after reconstructive surgery (Dai et al., 2017). *In vitro*, EVs derived from fetal mouse neural stem cells (NSCs) promote NSC differentiation through miR-9 targeting of *Hes1,* suggesting EVs could be used in conjunction with stem cell transplantation to treat neurodegenerative disease (Yuan et al., 2021). EVs also inhibit differentiation, for example, by preventing differentiation of pro-fibrotic myofibroblasts during inflammation, as discussed in Section 4.3 (Wang et al., 2017; Zhu et al., 2020).

Aging and disease attenuate the ability of EVs to influence differentiation, further highlighting the importance of this EV role during repair. For example, EVs from aged rat MSCs do not promote osteogenic differentiation or fracture healing as effectively as EVs from young rats, due to their enrichment for miR-128-3p, which targets *Smad5* to downregulate osteogenic BMP signaling (Xu et al., 2020). Xu et al. (2018) found that cigarette smoke extract induces upregulation of miR-21 in human bronchial epithelial cell EVs, which promote differentiation of bronchial fibroblasts into pro-fibrotic myofibroblasts. Inhibition of miR-21 reduces cigarette smoke-induced airway damage, fibrosis, and loss of pulmonary function in mice, hinting at a potential therapeutic strategy for human smokers with chronic obstructive pulmonary disease, in whom serum exosomal miR-21 is also elevated (Xu et al., 2018). In summary, EVs from multiple cell types can positively and negatively impact differentiation of stem and progenitor cells in mammalian regeneration models. These observations suggest that endogenously produced EVs may function similarly *in vivo*, and that EVs with differentiation-modulating activity could be utilized or engineered to promote tissue repair*.*

### 4.7 Summary

Several major conclusions can be drawn from the last 10–15 years of research. First, EVs modulate most, if not all, cell behaviors required for regeneration. Second, many EV cargo classes, but especially miRNAs and proteins, have been implicated in regeneration. Third, EV cargos control cell behaviors during regeneration at many levels by targeting signaling pathways, gene expression, oxidative stress, and diverse other molecular mechanisms in recipient cells. Fourth, disease and aging can dysregulate EV activities during regeneration. Fifth, the timing and selective targeting of EV cargo delivery are important, since the promotion or inhibition of some behaviors (e.g., apoptosis or proliferation) at the wrong time, or in the wrong cell types, would be detrimental. Sixth, the great number of studies demonstrating EVs’ pro-regenerative activity suggests that controlling EV production *in vivo*, or delivery of exogenously derived or engineered EVs, could be a therapeutically viable strategy for improving human regeneration.

## 5 Extracellular Vesicle Research in Other Established and Emerging Model Organisms With Varying Regenerative Capacities: Progress and Prospects

Despite significant progress in understanding the roles of EVs, considerable knowledge gaps remain. For example, although EVs derived from cultured stem cells promote regeneration, whether stem cells or terminally differentiated cells modulate EV biogenesis in response to tissue damage *in vivo* remains poorly studied, since few studies of EVs derived from damaged tissues have been conducted (rare examples are mentioned in Section 4.3 and Section 4.6). Additionally, mouse and rat are widely utilized human disease models, but their regenerative capacity (like that of humans) is limited relative to other animals (Bely and Nyberg, 2010; Iismaa et al., 2018). This discrepancy between the activity of cell culture-derived EVs in gain-of-function experiments and the limited regenerative ability of widely used rodent models raises critical questions. Are the pro-regeneration activities of culture-derived EVs an artefact of culture conditions or physiologically irrelevant concentrations of transplanted EVs? Or do EVs regulate regeneration in some animals, but in ways that have not been evolutionarily conserved in traditional rodent models like *M. musculus* and *R. norvegicus* (Bely and Nyberg, 2010)?

**FIGURE 5:**
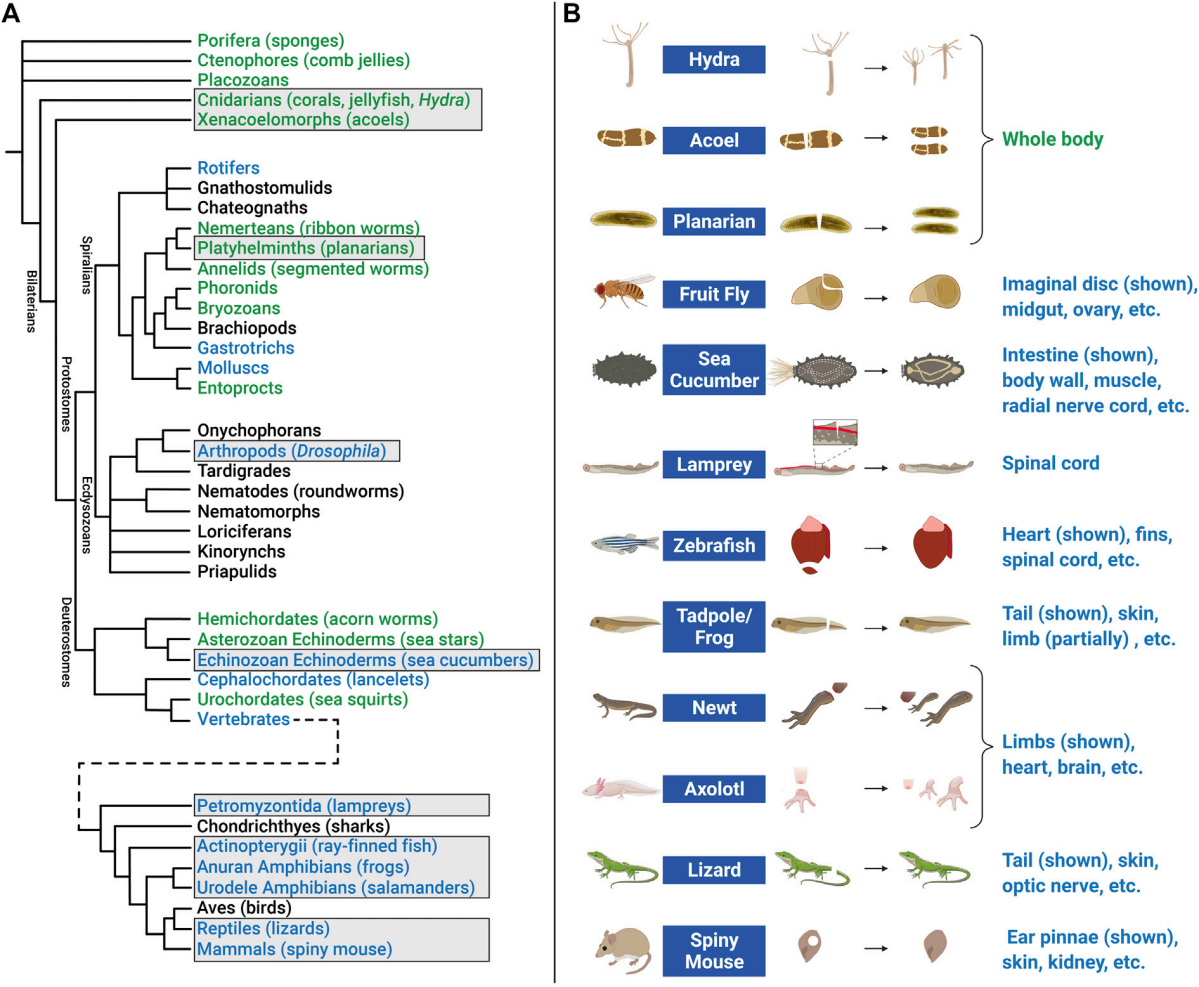
Animal models of regeneration. **(A)** Phylogenetic tree (cladogram) showing evolutionary relationships and degrees of regenerative capacity in animals. Evidence for regeneration within phyla is derived from previous reviews (Bely and Nyberg, 2010; Srivastava, 2021) and is indicated by color: whole body (green), structural (limb, organ, etc.) (blue), or no current evidence or tissue renewal only (black). Tree topology (branching) is based on multiple sources for Pre-bilaterians, Xenacoelomorphs, and Deuterostomes (Reich et al., 2015; Srivastava, 2021); Spiralians and Gnathifera (Marlétaz et al., 2019); Ecdysozoans (Giribet and Edgecombe, 2017); and Vertebrates (Bely and Nyberg, 2010). Cladogram branch length is schematized, and is not an estimate of relative time. Common names of representative animals in some phyla are listed in parentheses. Some clades have been omitted for simplicity. Examples of regeneration for clades in boxes are shown in the right panel. **(B)** Research animals that have greater regenerative abilities but have received less attention in EV research include hydra, acoel, planarian, fruit fly, sea cucumber, lamprey, zebrafish, tadpole/frog, newt, axolotl, lizard, and African spiny mouse. Non-exhaustive lists of some of the tissue(s) that these animals regenerate are indicated below each example image. Figure 5B created with BioRender.com.

**FIGURE 6:**
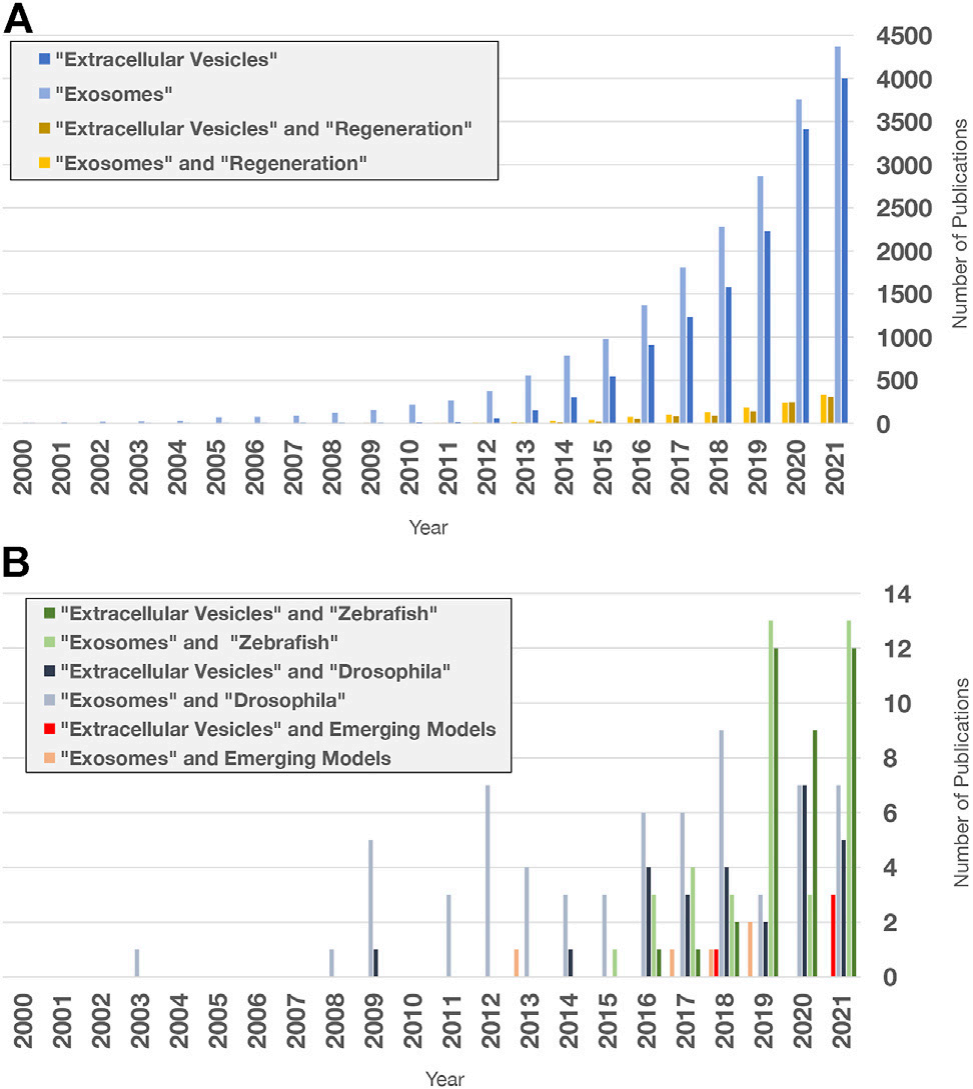
Extracellular vesicle and exosome research publications in PubMed. Search terms indicated were used to query “all fields” in PubMed for publication numbers since 2000 using the “Results by Year” tool. Only a fraction of publications focuses on regeneration and non-mammalian models. **(A)** Number of publications on EV and exosome research (blue), limited by Boolean “and” search for the term “regeneration” (yellow). **(B)** Number of publications containing the terms and organisms indicated. Publications with “acoel,” “hydra,” “planarian,” “sea cucumber,” “axolotl,” “newt,” “lamprey,” “lizard,” and “spiny mouse” were added together for the Emerging Models category. Publications with “RNA exosome” in any field were excluded.

Investigating EV roles in additional paradigms, such as digit tip regeneration (observed in Rhesus monkeys, young mice, and human children) and neonatal mouse heart regeneration, could help to address these questions (Porrello et al., 2011; Dolan et al., 2018; Del Campo et al., 2022). However, it may be necessary to extend studies to additional model organisms. For example, research in zebrafish (*D. rerio*) and the fruit fly *D. melanogaster* has contributed to our understanding of molecular mechanisms that control proliferation, differentiation, migration, apoptosis, and other cell behaviors that promote development, tissue renewal, and regeneration (Gilbert, 2017; Marques et al., 2019; Fox et al., 2020). In addition, organisms with greater regenerative capacity can be found across the Animal Kingdom (Figure 5A). Hydra, planarians, salamanders (axolotls and newts), lizards, African spiny mice, and other animals have become tractable research organisms because of the application of functional genomics (high throughput sequencing, RNA interference, genome editing, etc.) and other molecular methods (Sánchez Alvarado, 2018; Ivankovic et al., 2019; Joven et al., 2019; Jacyniak et al., 2017; Vogg et al., 2019; Maden and Varholick, 2020). These animals replace and reorganize cells in epithelial tissues, regenerate amputated fins and limbs, repair internal organs, and even regenerate whole bodies from tiny tissue fragments, completely restoring tissue morphology and function (Figure 5B). Investigations in these animals have begun to identify fundamental mechanisms and principles that define regenerative competence (e.g., the nature of early injury-induced signals, regeneration-specific gene expression and reprogramming, and cellular sources of new tissue) (Poss, 2010; Tanaka and Reddien, 2011; Niethammer, 2016; Zhao et al., 2016; Duncan and Sánchez Alvarado, 2019). Furthermore, comparative studies have begun to identify potential strategies for improving regenerative ability (e.g., targeting of inhibitory regulators and modulating inflammation) (Aguirre et al., 2014; Simkin et al., 2017). Although the number of publications focusing on EVs in regeneration has dramatically increased in recent years (Figure 6A), research organisms with greater regenerative capacity have received little attention (Figure 6B). In this last section, we highlight progress in understanding EV biology in two regeneration-competent models (zebrafish and *Drosophila*), and then discuss genomic and experimental evidence that EVs may regulate regeneration in other established and emerging research organisms. We conclude by suggesting the potential for these diverse models to accelerate EV research in several areas.

### 5.1 Zebrafish

Zebrafish regenerate multiple organs including fins, heart, retina, spinal cord, jaw, kidneys, pancreas, liver, and sensory hair cells (Gemberling et al., 2013; Marques et al., 2019). Depending on the tissue and type of damage, regeneration often occurs with either minimal scarring and/or eventual scar resolution (Becker et al., 1997; González-Rosa et al., 2011; Schnabel et al., 2011; Richardson et al., 2013). Upon injury, many cell types de-differentiate into lineage-restricted progenitor cells that re-enter the cell cycle, proliferate, and then differentiate to replace missing cell types (Jopling et al., 2010; Tu and Johnson, 2011; Stewart and Stankunas, 2012). After surgical amputation, fin regeneration occurs through formation of a blastema, a mass of tissue in which newly produced cells develop into new bone, muscle, blood vessels, and other tissues. After resection or cryoinjury to the heart ventricle, cardiomyocytes (CMs) de-differentiate and proliferate to replace damaged heart tissue [reviewed in Pronobis and Poss, 2020]. Similarly, after a variety of injuries to the retina, pluripotent adult retinal stem cells called Müller glia (MG) dedifferentiate into neuronal progenitor cells that give rise to different neuronal cell types that replace damaged cells [reviewed in Lahne et al., 2020]. The ability of zebrafish cells near damaged tissue to produce proliferative progenitors contrasts with injury responses in mouse and human, in which CMs and MG respond much less productively to injury.

Multiple studies using transgenic reporter lines to label EVs have demonstrated that zebrafish cells produce EVs, and that conserved proteins regulate their biogenesis. For example, EVs are produced by zebrafish cultured melanoma cells, apoptotic epithelial cells, and osteoblasts, and the yolk syncytial layer is a source of circulating EVs in the developing embryo whose secretion is Syntenin-dependent (Brock et al., 2019; Verweij et al., 2019; Didiano et al., 2020; Kobayashi-Sun et al., 2020; Mary et al., 2020). Several recent studies suggest that EVs may play a role during zebrafish regeneration. For example, using CD63-fluorophore transgenic reporters, Ohgo and colleagues demonstrated that EVs are present in blastemas of the regenerating caudal fin *in vivo*, and that these vesicles may be transferred between subcutaneous tissue and epidermis during regeneration (Ohgo et al., 2020). In another study, Scott et al. (2021) used cell-type-specific promoters to drive EV reporter expression, and showed that EVs are produced by both CMs and endothelial cells (EC-EVs). After myocardial cryoinjury, the number of EC-EVs decrease as a proportion of total EV number, and overall EV size is decreased, suggesting EV production may be modulated by injury in cell-specific ways (Scott et al., 2021). In an effort to determine whether EVs could functionally induce proliferation in the retina, Didiano et al. (2020) injected EVs from mammalian stem cells, iPSCs, and cancer cell lines into adult, undamaged retinas. EVs from C6 rat glioma cells increased proliferation of MG-derived cells to the greatest degree. The authors attributed the mechanism to the transcription factor Ascl1a, which is required for zebrafish retinal regeneration, because *ascl1a* expression increased after EV administration and *ascl1a* knockdown abolished EV-induced proliferation (Fausett et al., 2008; Didiano et al., 2020). Together, these studies suggest that 1) EVs are produced by a variety of zebrafish cells, including those in blastemas; 2) injury can alter EV production; and 3) EVs may upregulate transcriptional regulators required for reprogramming and regenerative proliferation. In the future, zebrafish is likely to contribute additional understanding of how EVs coordinate regeneration *in vivo*.

### 5.2 *Drosophila melanogaster*

Although adult fruit fly appendages and many organs are not capable of regeneration, some tissues do mount effective responses to damage and cell death. For example, resident stem cells in the adult midgut proliferate in response to cytotoxin-induced cell death, and germline stem cell daughters can de-differentiate to replace stem cells lost due to starvation or other stresses [reviewed in Fox et al., 2020]. Flies can also regenerate imaginal discs, epithelial pouches of cells in developing larvae that give rise to wings, eyes, and other structures in the adult (Hariharan and Serras, 2017; Ahmed-de-Prado and Baonza, 2018). In response to amputation, as well as more recent elegant genetic ablation approaches, imaginal discs regenerate through wound closure, proliferation, differentiation, and reprogramming of cellular identity (“transdetermination”) (Herrera and Morata, 2014; Hariharan and Serras, 2017).

*Drosophila* cells produce EVs, and conserved regulators likely function in their biogenesis. For example, cell lines derived from *Drosophila* tissues produce EVs carrying rRNA, mRNA, and numerous categories of small non-coding RNAs, as well as homologs of ALIX, TSG101, Rabs, Tetraspanins, and other EV-associated proteins (Koppen et al., 2011; Gross et al., 2012; Lefebvre et al., 2016). Functional EVs are also produced *in vivo:* male reproductive glands secrete EVs that inhibit female remating behavior, an activity that depends on both Alix and Rab11 (Corrigan et al., 2014). Although no studies directly link EVs to regeneration in *Drosophila,* several intriguing observations suggest EVs may be involved. Wingless (Wg), the *Drosophila* Wnt1 homolog, is upregulated in imaginal discs after amputation or genetic ablation, and is required for proliferation and growth (Gibson and Schubiger, 1999; McClure et al., 2008; Smith-Bolton et al., 2009; Katsuyama et al., 2015). Gross and colleagues found that Wg and its cargo receptor Evi/Wntless are secreted in EVs (labeled by transgenic expression of mammalian CD63) by imaginal disc cells during development, and identified the R-SNARE Ykt6 as a novel regulator of EV-mediated Wg secretion in an RNAi screen of EV-associated proteins (Gross et al., 2012). Similarly, Hedgehog (Hh) is another secreted morphogen that forms concentration gradients in imaginal discs and regulates cell fate changes during leg disc regeneration (Gibson and Schubiger, 1999; Beira and Paro, 2016). Gradilla et al. (2014) found that wing imaginal disc EVs transport Hh and its co-receptor Interference hedgehog (Ihog). They showed that Hh contained in EVs activates Hh-dependent transcription in cultured wing disc cells, and EV biogenesis regulators (e.g., Vps22, Vps24, sphingomyelinase, and Ykt6) are required for Hh secretion and full Hh gradient length *in vivo* (Gradilla et al., 2014). Together, these studies suggest that *Drosophila* EVs transport two morphogens on their surface that regulate growth and patterning of imaginal disc regeneration, and are capable of inducing signaling. However, whether EVs are required for intercellular communication during regeneration still remains unexplored. Powerful genetic tools and the speed with which *Drosophila* regenerates should lead to identification of additional mechanisms used by animals to control EV biogenesis and signaling during regeneration.

### 5.3 Emerging Regeneration Models

Studies in zebrafish and *Drosophila* demonstrate that mechanisms of EV biogenesis are broadly conserved, and that EVs are likely to function in tissue repair and regeneration, although their precise roles remain to be characterized. By contrast, few studies of EVs have been conducted in other animals with high regenerative capacity. In the future, studies in these organisms are likely to refine our understanding of how EVs function during regeneration for several reasons.

First, the same cell behaviors (survival, proliferation, etc.) modulated by EVs in mouse, fish, and flies also drive regeneration in these emerging models. In hydra and planarians, regeneration is driven by dedicated populations of pluripotent stem cells (Ivankovic et al., 2019; Vogg et al., 2019). In axolotls and newts, injury induces de-differentiation and proliferation of lineage-restricted progenitors, although species-specific differences exist (Joven et al., 2019). In spiny mice, proliferation and new tissue differentiation occur after a variety of injuries, but the cellular origins of new tissue remain to be fully elucidated (Maden and Varholick, 2020). Numerous studies have identified regeneration-associated cell behaviors in these organisms that underlie their greater regenerative capacity (Table 1). Many of these behaviors are not observed in poorly regenerating tissues in widely used rodent models (Poss, 2010; Zhao et al., 2016; Iismaa et al., 2018). For example, apoptotic cells secrete Wnt3 to drive regenerative proliferation in hydra, and spiny mice regulate ECM remodeling in specialized ways to achieve fibrosis- and scar-free regeneration after skin, kidney, heart, and spinal cord injury (Table 1). In such cases, interspecies differences in how EVs non-autonomously regulate apoptosis, mitogen transport, inflammation, and/or ECM remodeling could theoretically contribute to better regeneration.

**TABLE 1:** Cellular behaviors underlying regenerative capacity in emerging models.

Organism and Cell Behavior	Evidence of Regeneration-specific Control/Modulation	References
**Hydra**		
Apoptosis	Apoptotic cells at amputation site secrete pro-proliferative Wnt3a	Chera et al. (2009)
ECM Remodeling	ECM remodeling required for head regeneration	Shimizu et al. (2002)
Proliferation	Maintenance of dedicated stem cells that proliferate in response to injury	Hobmayer et al. (2012), Buzgariu et al. (2018)
Migration	Stem cell migration towards injury	Boehm and Bosch (2012)
Differentiation	Re-establishment of axial polarity (Wnt signaling) controls head/foot identity during new tissue differentiation	[reviewed in Vogg et al. (2019)]
**Planarians**		
Apoptosis/Survival	Differential control of neuronal survival in pre-existing and regenerating tissue	LoCascio et al. (2017), Karge et al. (2020)
Immunity/Inflammation	Disruption of innate immune signaling compromises regeneration	Arnold et al. (2016)
Proliferation	Maintenance of dedicated pluripotent stem cells (neoblasts) that proliferate in response to injury	Baguñà et al. (1989), Wenemoser and Reddien (2010)
Migration	Stem cell migration towards amputation site; remodeling/collective migration of pre-existing intestinal tissue in regenerating fragments	Forsthoefel et al. (2011), Guedelhoffer and Sánchez Alvarado (2012)
Differentiation	Re-establishment of axial polarity cue expression controls patterning and differentiation of regenerating tissue	[reviewed in Reddien (2018)]
**Axolotls and Newts**		
Cell death	Programmed cell death induces de-differentiation of myofibers to proliferative progenitors	Wang et al. (2015)
Immunity/Inflammation	Macrophages are required for regeneration	Godwin et al. (2013)
ECM/Fibrosis	Scar-free skin, limb, and organ regeneration	[reviewed in Godwin et al. (2014), Erickson and Echeverri (2018)]
Proliferation	Pro-proliferative MARCKS-like protein secreted in axolotl (but not mammals) to drive blastema formation; Schwann cell-expressed newt Anterior Gradient protein promotes proliferation in the limb blastema	Kumar et al. (2007), Sugiura et al. (2016)
De-differentiation	Injury-induced cell cycle re-entry of newt skeletal muscle myotubes and cardiomyocytes	Oberpriller and Oberpriller (1974), Tanaka et al. (1997), Bettencourt-Dias et al. (2003)
Differentiation	Maintenance of positional identity and re-establishment of axial polarity controls patterning and differentiation during limb regeneration	[reviewed in Vieira and McCusker (2019)]
Transdifferentiation	Newt pigmented epithelial cells transdifferentiate to regenerate lens	[reviewed in Henry and Tsonis (2010)]
**Spiny Mice**		
Immunity/Inflammation	Pro-regenerative M2 macrophages required for ear pinna regeneration; spatial restriction/reduction of pro-inflammatory macrophages during ear and skin regeneration	Brant et al. (2016), Simkin et al. (2017), Maden (2018), Brant et al. (2019)
ECM/Fibrosis	Fibrosis resolves in dorsal skin wounds and injured adult kidney; decreased collagen deposition during skin and spinal cord regeneration; upstream fibrosis-associated Wnt expression different between *A. cahirinus* and *M. musculus*	Seifert et al. (2012), Brant et al. (2016), Brant et al. (2019), Streeter et al. (2020), Okamura et al. (2021)
Proliferation	Elevated proliferation associated with skin and ear pinnae regeneration	Seifert et al. (2012), Maden (2018)
Differentiation	Repeated muscle differentiation after chronic injury	Maden et al. (2018)
**Additional examples**		
Acoels: Proliferation	Maintenance of dedicated pluripotent stem cells (neoblasts) that proliferate in response to injury	Srivastava et al. (2014)
Annelids: Migration	Stem cell migration towards amputation site	Zattara et al. (2016)
Sea cucumber: De-differentiation	Mesenterial muscle de-differentiation during digestive tract regeneration	Candelaria et al. (2006)
Lampreys: Migration	Axon regrowth, synapse regeneration, and full functional recovery after spinal cord transection	Rovainen (1976), Oliphint et al. (2010)
*Xenopus* tadpole: Apoptosis	Apoptosis is required for regeneration	Tseng et al. (2007)
Lizard: Proliferation	Proliferation of multiple cell types occurs during tail regeneration; homologs of proliferation-associated miRNAs upregulated during tail regeneration	[reviewed in Lozito and Tuan (2017)], Hutchins et al. (2016)

Second, bioinformatic searches of transcriptome data indicate that common EV markers and EV biogenesis regulators are conserved in these systems (Table 2 and Supplementary Table S1). For example, the genomes of representative species encode orthologs of TSG101, ALIX, Flotillin-1, Syntenin-1, and Rab-7a. In addition, homologs of the Tetraspanin CD63 are also present in the transcriptomes of each organism.

**TABLE 2:** Similarity of EV markers and biogenesis regulators between human and emerging models.

Human EV marker/Regulator^a^	Hydra (*Hydra vulgaris*)		Planarians (*Schmidtea mediterranea*)		Axolotl (*Ambystoma mexicanum*)		Spiny Mouse (*Acomys cahirinus*)	
	Identity (%)	*E* value	Identity (%)	*E* value	Identity (%)	*E* value	Identity (%)	*E* value
TSG101	39	1.39 × 10^−92^	38	1.63 × 10^−85^	84	0	94	0
Alix	47	2.28 × 10^−155^	38	1.94 × 10^−156^	75	0	95	0
Flotillin-1	61	4.44 × 10^−155^	61	8.98 × 10^−154^	82	0	97	0
Syntenin-1	52	1.24 × 10^−97^	51	3.29 × 10^−103^	84	0	90	0
Rab-7a	85	1.62 × 10^−127^	76	3.35 × 10^−117^	99	3.55 × 10^−155^	100	9.32 × 10^−154^
CD63	36	4.49 × 10^−24^	25^b^	3.27 × 10^−15^	79	1.16 × 10^−139^	76	1.94 × 10^−108^

a See Supplementary Table S1 for the transcript/protein ID of the top ortholog in each species.

b The top planarian CD63 homolog hit three human Tetraspanin-6 isoforms, followed by human CD63 in the reciprocal BLASTX query, suggesting high similarity, but a lack of one-to-one orthology in planarians.

Methods: Human TSG101 (NCBI NP_006283.1), Alix (NCBI NP_037506.2), Flotillin-1 (NCBI NP_005794.1), Syntenin-1 (NCBI NP_005616.2), Rab-7a (NCBI NP_004628.4), and CD63 (NCBI NP_001254627.1) proteins were used to query emerging model transcriptomes for orthologs using TBLASTN, or BLASTP (Axolotl). Presence of conserved protein domains in target sequences was verified using NCBI Conserved Domain Search, then Human RefSeq Protein was reciprocally queried with each top hit using BLASTX/BLASTP. All organisms’ top ortholog hit the corresponding human protein except for the top planarian CD63 hit. Amino acid identity and *E* values for these top orthologs are shown.

Databases: Hydra 2.0 Genome Project Portal (https://research.nhgri.nih.gov/hydra/)—Juliano Trinity (JT) assembly (Juliano et al., 2014); PlanMine (https://planmine.mpicbc.mpg.de)—Dresden dd_Smed_v6 assembly (Brandl et al., 2016); Axolotl Transcriptomics Database (https://portals.broadinstitute.org/axolotlomics/)—TransDecoder predicted protein sequences (Bryant et al., 2017) queried in Geneious Prime 2021.2.2; Spiny Mouse Sequence Server 2.0.0rc8 (spinymouse.erc.monash.edu/sequenceserver)—tr2aacds_v2 annotated protein assembly (Mamrot et al., 2017).

Third, hydra, newts, frogs, and sea cucumbers produce EVs. In hydra, EV-like particles were first superficially described in an ultrastructural study of gold nanoparticle trafficking (Marchesano et al., 2013). More recently, Moros and colleagues used ultracentrifugation to collect particles with EV-like size and morphology from hydra culture medium (Moros et al., 2021). Mass spectrometry analysis of these EVs revealed common cargo/biogenesis (CD63, Alix, and Syntenin) and signaling (Notch, NOD2) protein homologs, while RNA sequencing identified thousands of coding and non-coding RNAs, including multiple Wnt signaling pathway components. EV treatment of hydra induced Wnt3 expression, modestly increased the rate of head regeneration, and delayed foot regeneration, suggesting hydra EVs possess biological activity that can modulate regeneration (Moros et al., 2021). In newts, myogenic precursor cells secrete EV-like particles in culture that carry protein as well as coding and non-coding RNA (Middleton et al., 2018). Conditioned media from these cells protect rat CMs from apoptosis caused by oxidative stress, likely through upregulation of PI3K/Akt signaling (Middleton et al., 2018). Treatment of the newt cells with an EV biogenesis inhibitor reduces EV output and attenuated the conditioned media’s protective effect (Middleton et al., 2018). In addition to being an interesting example of EV-mediated interspecies communication (Ju et al., 2013; Mu et al., 2014), these results suggest that EVs with pro-regenerative activity are produced by newts. Finally, although roles in regeneration have not been investigated, EVs have been purified from both the frog *Xenopus laevis* and the sea cucumber *Stichopus japonicus,* further supporting the idea that EV-mediated communication is conserved across many animal regeneration models (Danilchik and Tumarkin, 2017; Jo et al., 2021).

### 5.4 Knowledge Gaps That Model Organisms Could Help to Address

Altogether, these observations suggest the likelihood that EVs promote recovery from tissue injury in established and emerging models of regeneration. Exploiting the genetic tools in zebrafish and fruit fly, and the growing set of molecular and genomic tools and high regenerative capacity of emerging models, could accelerate progress towards addressing several fundamental questions.

#### 5.4.1 How is Extracellular Vesicle Biogenesis Regulated?

As we have outlined above, the genomes of regeneration models encode many known markers and regulators of EV biogenesis. With a few exceptions (Syntenin in zebrafish, Alix and Ykt6 in flies), however, the molecular requirements for EV secretion are almost entirely unknown (Gross et al., 2012; Corrigan et al., 2014; Verweij et al., 2019). Methodical testing of known regulators will help to clarify which mechanisms are conserved across animal phyla. Most known biogenesis regulators possess additional functions in endocytosis, endosomal trafficking, exocytosis, cytokinesis, and other intracellular processes, but few (if any) molecules with dedicated roles in EV biogenesis have been identified (van Niel et al., 2018). Genetic screens and other strategies in additional models could therefore help to identify more specialized regulators with *in vivo* relevance, and to distinguish constitutive, tissue-specific, and regeneration-specific roles.

#### 5.4.2 Does Injury Modulate Extracellular Vesicle Biogenesis?

The number, size, and/or composition of EVs can be affected by tissue damage. For example, plasma EV numbers increase and EV composition (based on cellular origin) is altered in human trauma patients (Kuravi et al., 2017). In mice, hepatic ischemia/reperfusion injury increases the number of circulating EVs that promote regenerative proliferation (Nojima et al., 2016). By contrast, spinal cord injury decreases circulating EVs overall, while increasing the CD81-positive subpopulation and altering miRNA content (Khan et al., 2021). Notwithstanding these studies, a comprehensive understanding of this phenomenon across tissues and organisms is lacking, and there is limited understanding of mechanisms by which EV output is controlled. Phosphorylation of EV biogenesis regulators is one possible mechanism: pyruvate kinase type M2 can promote EV release through phosphorylation of SNAP-23, while the phosphatase Shp2 inhibits EV release through dephosphorylation of Syntenin, but it is not clear whether these mechanisms are relevant during regeneration (Wei et al., 2017; Zhang et al., 2021b). Although continued development of methods to isolate EVs and monitor their local production in regions of tissue damage *in vivo* will be needed (Brock et al., 2019; Verweij et al., 2019), investigations in emerging models will expand our understanding of how injury is transduced into changes in EV output.

#### 5.4.3 Which Cargos Promote Cell Behaviors Required for Successful Regeneration?

EVs in regeneration models are likely to carry some of the same cargos that promote repair in mouse, and they may transport secreted cues like Wnts and Hh proteins already known to modulate regeneration in *Drosophila* and other animals (Gross et al., 2012; Gradilla et al., 2014). Given that injury also causes upregulation of many cytoplasmic proteins, mRNAs, and non-coding RNAs in emerging models [e.g., González-Estévez et al., 2009; Rao et al., 2009; Holman et al., 2012; Monaghan et al., 2012; Wenemoser et al., 2012; Krishna et al., 2013; Sasidharan et al., 2013; Brant et al., 2015; Petersen et al., 2015; Hutchins et al., 2016; Ong et al., 2016; Yoon et al., 2020], it is tempting to speculate that some of these may function in currently unappreciated ways as EV cargo, and therefore that many more secreted modulators of regeneration remain to be identified. Efforts to identify novel regeneration-specialized cargo may require methodical EV characterization over regeneration time courses, and development of methods to selectively control EV cargo loading in newer research organisms. Work in emerging models could provide a more comprehensive view of cargo identity, loading, and delivery that might speed comparative studies and translational efforts.

#### 5.4.4 What Are the Cellular Sources of Extracellular Vesicles During Regeneration?

Although many studies demonstrate that cultured mouse and human stem cells are a significant source of EVs, investigations of whether lineage-restricted progenitor cells or fully differentiated cells produce EVs are rare, possibly due to the greater difficulty of culturing post-mitotic cells, and limited tools for tracking and purifying EVs from specific cell types *in vivo*. Nonetheless, the fact that zebrafish CMs, *Drosophila* reproductive gland cells, and cultured newt muscle cells produce EVs (Corrigan et al., 2014; Scott et al., 2021) suggests that committed cell types could also produce EVs during regeneration. Intriguingly, apoptotic cells secrete Wnt3 to promote proliferation during *Hydra* regeneration (Chera et al., 2009), and dying zebrafish epithelial stem cells secrete Wnt8a on apoptotic bodies that promote proliferation of neighboring stem cells (Brock et al., 2019). Mammalian cells release apoptotic bodies (a class of MV) by blebbing at the PM, and may produce apoptotic exosome-like vesicles (“ApoExos”) derived from the endosomal pathway (Kakarla et al., 2020). Thus, two important challenges in emerging models will be to determine whether injury alters EV output by stem, progenitor, and/or committed cell types, and whether dying cells, far from being just a detrimental consequence of tissue damage, also provide pro-regenerative instructions through EV secretion.

#### 5.4.5 Do Extracellular Vesicles Modulate Early Injury Responses?

Tissue injury induces changes in gene expression, cell states, inflammation, and other processes, often within a few minutes to a few hours. Many of these processes require extensive genomic reprogramming, and are thought to be initiated, in part, by growth factor receptor signaling and intracellular kinase cascades (Fraguas et al., 2011; Almuedo-Castillo et al., 2014; Owlarn et al., 2017; Duncan and Sánchez Alvarado, 2019; Srivastava, 2021). However, we lack a comprehensive mechanistic explanation for how regenerative programs are initiated. Although various damage-inducing stresses (e.g., irradiation, cisplatin treatment, hypoxia) can increase EV output within 24 h, only a few studies have focused on whether EV biogenesis can respond to external stimuli more rapidly (Lehmann et al., 2008; King et al., 2012; Xiao et al., 2014; Beer et al., 2015). In one study, treatment with inducers of endoplasmic reticulum stress promoted MVB formation and upregulated EV secretion within 3 h (Kanemoto et al., 2016). In another study, stimulation of the histamine H1 G-protein coupled receptor induced MVB-PM fusion and CD63-positive EV release within 60 s (Verweij et al., 2018). These observations suggest that EV biogenesis could theoretically respond to tissue damage quickly enough to influence the earliest cellular and molecular events during regeneration. Exploring this potential role for EVs is therefore another intriguing avenue for further investigation.

## 6 Conclusion

Over the past 15–20 years, we have witnessed an expansion of research into the roles of EVs in regeneration, which parallels the stunning growth of the field of EV biology more generally. Simultaneously, the field of regeneration has been transformed by the rapid development of animals with high regenerative capacity into tractable organisms amenable to genomic, molecular, and cellular investigation. Research at the intersection of these two frontiers promises new insights into how intercellular communication coordinates cellular behaviors during regeneration, and will accelerate progress towards regenerative medicine’s ultimate goal: improving human health.
