# Supplementary material for: An Emerging Frontier in Intercellular Communication: Extracellular Vesicles in Regeneration
Source: Front Cell Dev Biol. 2022 May 11;10:849905. doi: 10.3389/fcell.2022.849905 (PMC9130466; doi:10.3389/fcell.2022.849905)
Supplement: Supplementary file 1 [file Table1.DOCX]

**Supplementary Table 1.** Transcript or protein ID of top EV marker and biogenesis regulator orthologs in emerging regeneration models. Related to Table 2.

| **EV Marker/Regulator^†^** | **Hydra**  **(*Hydra vulgaris*)** | **Planarians**  **(*Schmidtea mediterranea*)** | **Axolotl**  **(*Ambystoma mexicanum*)** | **Spiny mouse**  **(*Acomys cahirinus*)** |
| --- | --- | --- | --- | --- |
| **TSG101** | JT_TRINITY_DN26613_c0_g1_i1 | dd_Smed_v6_1686_0_1 | c1022617_g1_i1\|m.72245 | Locus_60542 |
| **Alix** | JT_TRINITY_DN19235_c0_g1_i1 | dd_Smed_v6_1373_1_2 | c1079286_g1_i1\|m.198357 | Locus_326540 |
| **Flotillin-1** | JT_TRINITY_DN22849_c0_g1_i3 | dd_Smed_v6_1637_0_1 | c1072423_g1_i1\|m.153034 | Locus_561867 |
| **Syntenin-1** | JT_TRINITY_DN10653_c0_g1_i1 | dd_Smed_v6_387_0_1 | c1084797_g1_i1\|m.243732 | Locus_129705 |
| **Rab-7a** | JT_TRINITY_DN11408_c0_g1_i1 | dd_Smed_v6_491_0_1 | c1071179_g1_i1\|m.146656 | Locus_328585 |
| **CD63** | JT_TRINITY_DN25727_c0_g1_i1 | dd_Smed_v6_2452_0_1 | c1511502_g1_i1\|m.354373 | Locus_545837 |

^†^See Table 2 for amino acid identity and *E* value similarity score for each ortholog.
